# Ufd2p synthesizes branched ubiquitin chains to promote the degradation of substrates modified with atypical chains

**DOI:** 10.1038/ncomms14274

**Published:** 2017-02-06

**Authors:** Chao Liu, Weixiao Liu, Yihong Ye, Wei Li

**Affiliations:** 1 State Key Laboratory of Stem Cell and Reproductive Biology, Institute of Zoology, Chinese Academy of Sciences, Beijing 100101, China; 2 Laboratory of Molecular Biology, National Institute of Diabetes and Digestive and Kidney Diseases, National Institutes of Health, Bethesda, Maryland 20892, USA

## Abstract

Ubiquitination of a subset of proteins by ubiquitin chain elongation factors (E4), represented by Ufd2p in *Saccharomyces cerevisiae*, is a pivotal regulator for many biological processes. However, the mechanism of Ufd2p-mediated ubiquitination is largely unclear. Here, we show that Ufd2p catalyses K48-linked multi-monoubiquitination on K29-linked ubiquitin chains assembled by the ubiquitin ligase (Ufd4p), resulting in branched ubiquitin chains. This reaction depends on the interaction of K29-linked ubiquitin chains with two N-terminal loops of Ufd2p. Only following the addition of K48-linked ubiquitin to substrates modified with K29-linked ubiquitin chains, can the substrates be escorted to the proteasome for degradation. We demonstrate that this ubiquitin chain linkage switching reaction is essential for ERAD, oleic acid and acid pH resistance in yeast. Thus, our results suggest that Ufd2p functions by switching ubiquitin chain linkages to allow the degradation of proteins modified with a ubiquitin linkage, which is normally not targeted to the proteasome.

## Abstract (web summary)

How ubiquitination affects the proteins it modifies varies according to the type of linkage between ubiquitin moieties. Here, Liu *et al*. show how yeast Udf2p promotes K48 linkage formation onto K29-linked chains to generate branched K29-K48 ubiquitin chains that target its substrate to the proteasome.

Protein ubiquitination is an important post-translational modification in which ubiquitin, a 76-residue protein, is covalently attached to target proteins. Ubiquitination requires three classes of enzymes that act in concert: a ubiquitin-activating enzyme (E1) forms a thioester bond between itself and the C-terminus of ubiquitin, a ubiquitin-conjugating enzyme (E2) receives the activated ubiquitin from the E1 by transthiolation, and a ubiquitin ligase (E3), including really interesting new gene (RING), homologous to E6-associated protein C-terminus (HECT) and RING-in-between-RING (RBR) type ligase, that transfers ubiquitin from the E2 to a lysine (K) or a serine/threonine residue in the target protein^1^,^2^,^3^,^4^. Ubiquitination regulates several cellular processes, including protein quality control, cell-cycle progression, endocytosis, cell signalling, autophagy, transcriptional regulation and DNA damage responses^1^,^2^,^5^,^6^,^7^. The functional diversity of ubiquitination is achieved by the ability of ubiquitin to form different types of conjugates, each representing a distinct functional signal^8^,^9^. In addition to monoubiquitination and multi-monoubiquitination, polyubiquitination involves linking ubiquitin to one of the seven lysine residues or to the N-terminal methionine (Met1) of ubiquitin itself^10^,^11^,^12^. Ubiquitin can also be conjugated simultaneously to more than one lysine residue within a ubiquitin moiety to form branched ubiquitin chains^13^,^14^,^15^.

Although several general models have been proposed to explain the mechanisms underlying linkage-specific polyubiquitination, the precise mechanisms underlying many polyubiquitin chain assembly reactions remain unclear, particularly those synthesizing the recently discovered atypical ubiquitin chains or branched chains^11^,^14^,^16^,^17^,^18^,^19^,^20^,^21^,^22^,^23^,^24^,^25^,^26^,^27^. In addition to the three enzyme classes mentioned above, a class of chain elongation factor was also indentified more than ten years ago, termed the E4 or ubiquitin elongation enzyme^19^,^28^. *In vitro*, E4s can promote ubiquitin transfer from an E2 to a substrate in a manner similar to the E3 enzyme, and E4s may act as a class of enzymes that preferentially use ubiquitin as a substrate^28^,^29^,^30^. Several models have been proposed to explain the mechanism of E4-mediated polyubiquitination. These include: (1) E4 might alter E2–E2 interaction or an E3–E2 complex to limit ubiquitin chain hydrolysis from the active site of either E2 or a HECT domain-containing E3; (2) E4 might act as a processivity-promoting factor by decreasing the rate of whole-chain transfer to substrate relative to the rate of chain elongation on E2 or E3 (ref. 16).

The first E4 enzyme discovered—Ufd2p— is a *Saccharomyces cerevisiae* protein previously implicated in the ubiquitin fusion degradation (UFD) pathway, where it degrades substrates bearing an N-terminal ubiquitin fusion^19^,^28^,^31^. In addition to the UFD pathway, Ufd2p has also been implicated in the ER-associated protein degradation (ERAD) process, where it interacts with the dislocation driving ATPase Cdc48p, and appears to act downstream of Cdc48p to increase the degree of ubiquitination. The protein also simultaneously facilitates the transfer of ERAD substrates to Rad23p through an interaction between its N-terminal region and the Rad23p ubiquitin-like domain^32^,^33^,^34^,^35^. The subsequent disassembly of the Ufd2p–Rad23p complex allows Rad23p to bind to the proteasome and promote substrate degradation^36^. In addition, deletion of *UFD2* partially stabilizes the transcription factor, Spt23p, in the OLE pathway, which controls membrane fluidity by regulating the expression of the Δ9 fatty acid desaturase *OLE1* (ref. 37). The crystal structure of Ufd2p has an elongated shape with a flexible C-terminal U-box domain structurally related to the RING domain found in most E3 ubiquitin ligases^30^. This domain is essential for the function of Ufd2p. However, it remains unclear how Ufd2p acts to promote the ubiquitination and degradation of diverse substrates. In the UFD pathway, Ufd2p acts in conjunction with Ufd4p, a HECT domain ubiquitin ligase^19^,^30^. The demonstrated E4 activity suggested that it might promote substrate degradation by elongating ubiquitin chains on a short ubiquitin chain synthesized by Ufd4p to meet the minimal chain length requirement for the proteasome. However, how precisely these two enzymes cooperate to lead to competent degradation is unclear.

Here, we show that Ufd2p reshapes a degradation signal by catalysing K48-linked multi-monoubiquitination on K29-linked ubiquitin chains conjugated to a UFD substrate, resulting in branched ubiquitin chains. These branched ubiquitin chain modifications are detected in the UFD substrate both *in vitro* and *in vivo*. This reaction is dependent on an interaction between the K29-linked ubiquitin chain of a substrate and the two N-terminal loops of Ufd2p. Importantly, it is only after addition of K48-linked ubiquitin conjugates by Ufd2p, that substrates modified by the K29-linked ubiquitin chains can be escorted by Rad23p, Dsk2p or Rpn10p for targeting to the proteasome. We show that the linkage switching by Ufd2p is essential for ERAD, oleic acid and acid resistance in budding yeast. We propose that Ufd2p functions to switch ubiquitin chain linkages rather than as a chain elongator, which transforms a non-degradable ubiquitin signal into one that is favored by the proteasome.

## Results

### Ufd4p and Ufd2p synthesize K29- and K48-linked ubiquitin chains

**Figure 1:**
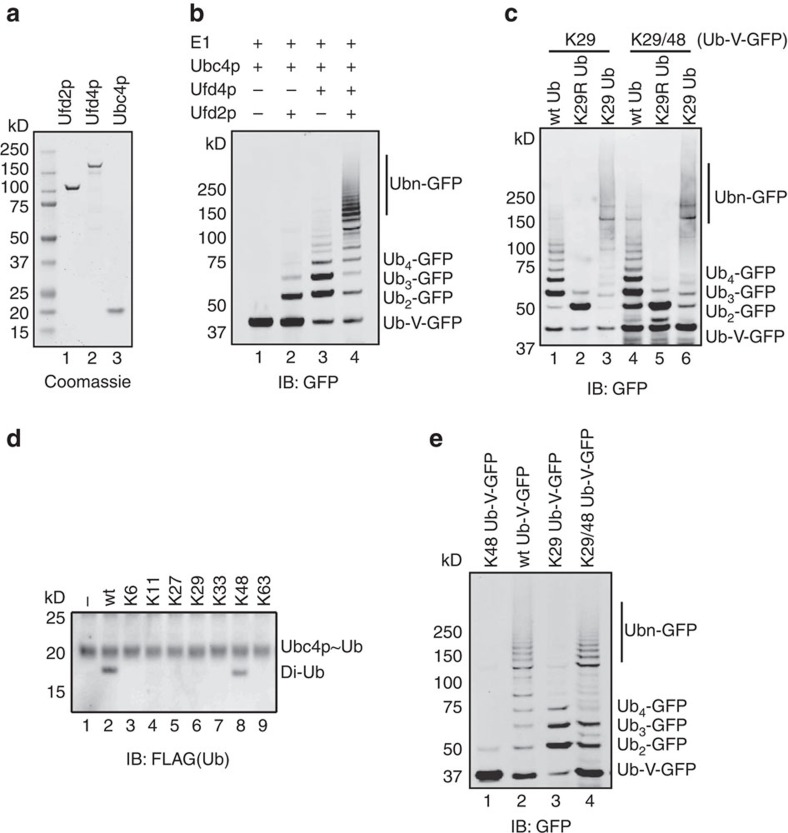
Ufd4p and Ufd2p synthesize K29- and K48-linked ubiquitin chains, respectively. (**a**) Coomassie blue-stained gels showing the expression and purification of relevant enzymes. (**b**) Reconstitution of the Ufd4p-Ufd2p ubiquitination system. Either Ufd2p (Lane 2), Ufd4p (Lane 3), or both (Lane 4) were added to the Ub-V-GFP ubiquitination system *in vitro*, which contains E1, E2, ubiquitin, ATP and Ub-V-GFP (Lane 1). (**c**) Ufd4p catalyses K29-linked polyubiquitin chain assembly on Ub-V-GFP. Ufd4p-mediated polyubiquitination of a Ub-V-GFP mutant with only K29 and K48 in its ubiquitin region (Lanes 4–5). The reaction products were indistinguishable from the products of the K29-only Ub-V-GFP mutant (Lanes 1–3) in the presence of wild-type, K29R- or K29-only ubiquitin. (**d**) The linkage specificity of Ufd2p-mediated ubiquitination was examined using a single-round ubiquitin turnover assay. K29R ubiquitin was charged onto the active cysteine of Ubc4p, and the reaction was quenched by adding NEM/EDTA. Wild-type ubiquitin or the K6-, K11-, K27-, K29-, K33-, K48-, or K63-only ubiquitin mutants (Lanes 2–9) was added in excess (10 fold of K29R ubiquitin) to the mixtures, together with Ufd2p, allowing transfer of the charged K29R ubiquitin to either wild-type ubiquitin or its variants to form free di-ubiquitin chains (Lanes 2 and 8). (**e**) K29 and K48 on Ub-V-GFP are necessary and required for full ubiquitination by Ufd4p and Ufd2p. K48-only Ub-V-GFP, wild-type Ub-V-GFP, K29-only Ub-V-GFP, and K29/48-only Ub-V-GFP were tested for their ability to be ubiquitinated by the Ufd4p-Ufd2p ubiquitination system. See also Supplementary Figs 1–3.

Ufd2p was shown to promote the polyubiquitination of a UFD substrate together with the ubiquitin ligase Ufd4p (refs 19, 28, 38). To study the mechanism of Ufd2p-catalysed polyubiquitination, we wanted to reconstitute the Ufd4p-Ufd2p-mediated ubiquitination *in vitro* using purified components (Fig. 1a,b). We first performed an E2 screening, which identified Ubc4p as the appropriate E2 for polyubiquitination by Ufd4p and Ufd2p (Supplementary Fig. 1). When purified Ufd2p was added to a reaction containing E1, Ubc4p and Ufd4p, it could efficiently enhance Ufd4p-mediated polyubiquitination of Ub-V-GFP (Fig. 1b), as previously demonstrated^19^,^38^.

To determine the linkage preference of the Ufd4p-Ufd2p system, we created a collection of Ub-V-GFP mutants that carry a single lysine in the ubiquitin moiety or have one of the seven lysine residues in ubiquitin mutated to arginine (K to R). *In vitro* ubiquitination using these Ub-V-GFP mutants showed that both K29 and K48 were required for Ufd4p and Ufd2p-mediated polyubiquitination of Ub-V-GFP because neither K48R, K29R nor K29, K48 single lysine Ub-V-GFP mutants could be efficiently polyubiquitinated when compared with wild-type (WT) Ub-V-GFP (Supplementary Fig. 2a,b).

To delineate the linkage specificity for each of the two enzymes, we first reconstituted the ubiquitination reaction using Ufd4p in the absence of Ufd2p. We found that in the Ufd4p-mediated polyubiquitination of Ub-V-GFP, K29 but not K48 on Ub-V-GFP was required (Supplementary Fig. 2c,d). This was because the ubiquitination pattern of Ub-V-GFP bearing only K29 and K48 was similar to that of the K29-only Ub-V-GFP mutant regardless of whether WT, K29R- or K29-only ubiquitin mutants were used in the reaction (Fig. 1c). In addition, the K29-linked polyubiquitin chain-binding protein Ufd3p (ref. 39) could efficiently pull down Ufd4p synthesized Ubn-GFP (Supplementary Fig. 3a). Thus, we concluded that the K29 of ubiquitin is preferentially used by Ufd4p to build polyubiquitin chains on Ub-V-GFP, in line with previous findings^19^,^31^,^40^.

To examine the linkage specificity of Ufd2p-mediated ubiquitination, we used a previously established single-round ubiquitin turnover assay^41^. FLAG-tagged ubiquitin was charged onto Ubc4p. The charging reaction was quenched by addition of N-ethylmaleimide (NEM)/Ethylene diamine tetraacetic acid (EDTA) and mixed with Ufd2p together with an excess amount (10-fold) of WT ubiquitin or ubiquitin mutants carrying only a single lysine as an acceptor. If the charged FLAG-ubiquitin was transferred to the added ubiquitin or its variants, it would result in the formation of unanchored free di-ubiquitin molecules. As shown in Fig. 1d, only WT ubiquitin and the K48-only ubiquitin mutant were able to accept ubiquitin from Ubc4p to form di-ubiquitin, suggesting that Ufd2p preferentially forms K48-linked ubiquitin chains. In further support of this notion, we found that the UFD-related deubiquitinase Otu1p, which hydrolyses K48-linked polyubiquitin chains but not K29-linked chains^33^,^42^, could remove the high molecular weight ubiquitin chains formed by Ufd2p and Ufd4p, but it could not hydrolyse short ubiquitin chains, which were presumably formed by Ufd4p (Supplementary Fig. 3b). Collectively, these results confirmed the previous findings that Ufd2p synthesized K48-linked ubiquitin chains^31^,^38^,^40^. Finally, the observation that the ubiquitination pattern of the K29/48-only Ub-V-GFP mutant was similar to that of WT Ub-V-GFP (Fig. 1e) further suggests that Ufd4p-Ufd2p mainly catalyses K29 and K48 mixed ubiquitin chains on the substrate.

### Ufd2p catalyses ubiquitination independent of Ufd4p

**Figure 2:**
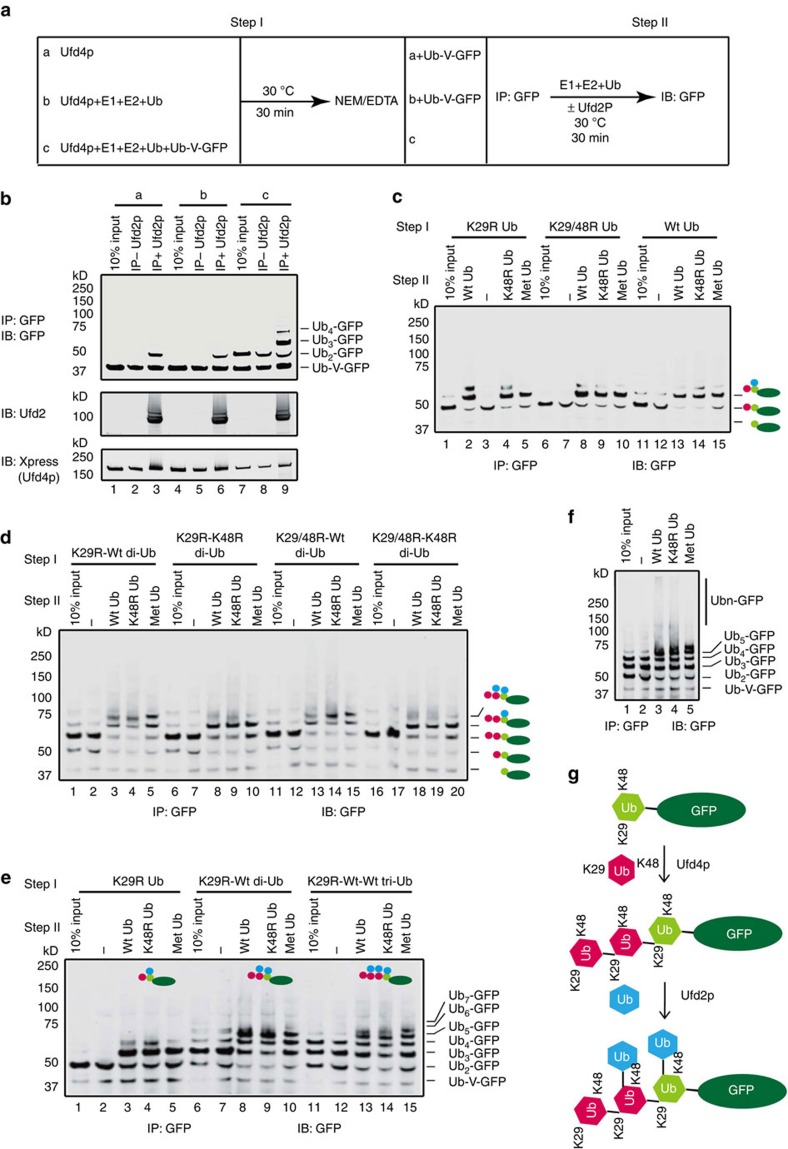
Ufd2p and Ufd4p synthesize branched ubiquitin chains on the substrate. (**a**) Schematic representation of the stepwise ubiquitination experiment. (**b**) Ufd2p promotes ubiquitination independent of Ufd4p. As illustrated in (**a**), either Ub-V-GFP or Ub2-GFP synthesized by Ufd4p was subjected to immunoprecipitation (IP) with the anti-GFP antibody. E1, E2, ubiquitin, ATP and Ufd2p were then added to the mixture. The reaction products were detected using the anti-GFP antibody; the Ufd2p and Ufd4p proteins were detected using the anti-Ufd2 and anti-Xpress antibodies, respectively. Input was from step I reaction products. To exclude the influence of Ufd4p in the second step, we used two controls: one contained Ufd4p alone (set a), and the other did not include Ub-V-GFP in step I (set b). (**c**) Ufd2p transfers one ubiquitin to the proximal ubiquitin of Ub2-GFP to form branched ubiquitin chains on the substrate. As illustrated in (**a**), K29R Ub, K29/48R Ub or wild-type Ub was used in step I, and wild-type Ub, K48R Ub or methylated Ub was used in step II. Input was from step I reaction products. (**d**) As in (**c**), except that the ubiquitin mutants in step I were replaced by K29R-Wt di-Ub, K29R-K48R di-Ub, K29/48R-Wt di-Ub, or K29/48R-K48R di-Ub. (**e**) As in (**c**), except that the ubiquitin mutants in step I were replaced with K29R Ub, K29R-Wt di-Ub and K29R-Wt-Wt tri-Ub. (**f**) As in (**e**), K29R-Wt-Wt tri-Ub was used in step I and wild-type Ub, K48R Ub or methylated Ub was used in step II, but the incubation time was 2 h. (**g**) A model for the functional role of Ufd2p and Ufd4p in the stepwise ubiquitination assay. First, Ufd4p assembles K29-linked ubiquitin chains on Ub-V-GFP; Ufd2p then catalyses multi-monoubiquitination on Ub-V-GFP modified with K29-linked ubiquitin chains to form branched ubiquitin chains. See also Supplementary Figs 4–6.

Because both Ufd4p and Ufd2p have E3 ligase activity^19^,^30^,^31^, we asked how Ufd2p cooperates with Ufd4p to extend oligo-ubiquitin chains. Conceptually, the two enzymes can work either cooperatively or sequentially. In a cooperative model, the two proteins may form a complex together with the E2 enzyme Ubc4p. Alternatively, a substrate that has been ubiquitinated by Ufd4p may engage Ufd2p independently of Ufd4p, allowing addition of K48-linked ubiquitin conjugates to the substrate^30^. To distinguish between these possibilities, we developed a stepwise ubiquitination assay (Fig. 2a–c). First, we incubated Ub-V-GFP with E1, Ubc4p, Ufd4p, ubiquitin and ATP to generate a short ubiquitin chain on Ub-V-GFP (Fig. 2b, Lane7). The reaction was then quenched by NEM/EDTA, which modifies the catalytic cysteine of Ufd4p and Ubc4p, inactivating and eliminating them from subsequent reactions. We then purified Ub-V-GFP and its modified forms using immunoprecipitation (IP) with an anti-GFP antibody. Immunoprecipitated materials were then incubated with E1, Ubc4p, Ufd2p, ubiquitin and ATP. The reaction was analysed by immunoblotting with an anti-GFP antibody. The results showed that under this experimental condition, Ufd2p transferred one ubiquitin to Ub-V-GFP with a low-efficiency in the absence of Ufd4p (Fig. 2b, Lanes 3, 6). However, once Ub-V-GFP was modified with a ubiquitin moiety by Ufd4p, most mono-ubiquitinated Ub-V-GFP (Ub2-GFP) could be switched to Ub3-GFP by Ufd2p (Fig. 2b, Lane 9 and Supplementary Fig. 6a). Because immunoblotting showed that trace amounts of Ufd4p were still present in the second reaction, to exclude the possibility that inactive Ufd4p might influence Ufd2p activity by a dominant-negative mode, we added extra Ufd4p, which was inactivated by NEM/EDTA to the second reaction. We found that the presence of inactive Ufd4p did not affect the efficiency of Ufd2p (Supplementary Fig. 4a). Moreover, addition of inactivated Ufd4p to the Ufd2p-mediated Ub-V-GFP ubiquitination system affected neither Ub-V-GFP mono-ubiquitination nor free ubiquitin chain formation (Supplementary Fig. 4b). The efficiency of Ub3-GFP production was also not affected by the addition of fully active Ufd4p in the second reaction or when K29R ubiquitin was used in the second reaction (Supplementary Fig. 4c). To further demonstrate that the stepwise reaction faithfully recapitulates the physiological condition, we used Ufd4p together with FLAG-K29R Ub to synthesize Ub2-GFP. Ub2-GFP was then purified via immunoprecipitation with anti-FLAG M2 Affinity Gel, and eluted by FLAG peptides under the native conditions (Supplementary Fig. 4d). The purified Ub2-GFP was then used as the substrate to test the ubiquitin transfer efficiency of Ufd2p in the presence or absence of the Ufd4p condition. As shown in Supplementary Fig. 4e, the presence of Ufd4p in the second reaction could not enhance the production of Ub3-GFP, and the ubiquitin transfer efficiency of Ufd2p in the stepwise reaction was almost the same as the normal *in vitro* ubiquitination condition (Supplementary Fig. 4f). Moreover, further incubation of ubiquitinated Ub-V-GFP after the second reaction with Ufd4p, E1, E2, ubiquitin and ATP did not result in further addition of ubiquitin conjugates (Supplementary Fig. 4g, h). This suggests that Ufd4p cannot act on substrates modified by Ufd2p. Altogether, these results suggest that Ufd2p and Ufd4p function in sequence to assemble ubiquitin chains on Ub-V-GFP, with Ufd4p acting first to prime a substrate with a K29-linked ubiquitin chain. Ufd2p subsequently works independently to add additional ubiquitin moieties.

### Ufd2p and Ufd4p synthesize branched ubiquitin chains

To dissect the topology of the ubiquitin chains synthesized by Ufd4p and Ufd2p, we used the K29R ubiquitin mutant in step I (Fig. 2a). This allowed us to specifically assemble K29-linked Ub2-GFP (Fig. 2c, Lane1). After purification, this Ub2-GFP species was used as the substrate for Ufd2p in the second step. We found that Ufd2p could efficiently add one ubiquitin to Ub2-GFP to form Ub3-GFP (Fig. 2c, Lanes 2, 4, 5), regardless of whether WT ubiquitin, the K48R mutant (K48R Ub) or methylated ubiquitin (Met-Ub) was used in the second reaction. Since ubiquitin could be added to either the distal or proximal ubiquitin on Ub-V-GFP, to distinguish between these possibilities, we replaced WT ubiquitin with the K29/48R ubiquitin mutant in step I. Because Ufd2p preferentially added ubiquitin to the K48 of ubiquitin, mutant ubiquitin should block chain extension by Ufd2p (if Ufd2p only extend the chain from the distal end). However, this substitution did not affect the addition of ubiquitin to Ub2-GFP by Ufd2p (Fig. 2c, Lanes 8–10 compared with Lanes 2, 4, 5), suggesting that Ufd2p does not extend the oligo-ubiquitin chain formed by Ufd4p from the distal end. Intriguingly, even when WT Ub was used in the first step, only a single ubiquitin moiety was transferred onto Ub2-GFP (Fig. 2c, Lanes 13–15). Thus, it appears that Ufd2p may preferentially add ubiquitin to the proximal ubiquitin of Ub2-GFP, resulting in the formation of branched ubiquitin chains (Supplementary Fig. 5a,b).

Next, we examined whether the proximal end preference is applicable to a longer ubiquitin chain on Ub-V-GFP. To this end, we synthesized and purified K29-linked di- or tri-ubiquitin chains (Supplementary Fig. 5) via Ufd4p-mediated polyubiquitination (see Methods)^43^. If di-ubiquitin carrying a UbK29R mutant at the distal end was used in step I to synthesize Ub3-GFP (Fig. 2d, Lane 1), Ufd2p transferred only two ubiquitin moieties to Ub3-GFP regardless of whether WT Ub, K48R Ub or Met-Ub was used in step II (Fig. 2d, Lanes 3–5; Supplementary Fig. 5c). Since a di-ubiquitin bearing a K29R and K48R double mutant at the distal site yielded a similar result (Fig. 2d, Lanes 13–15), the distal ubiquitin is apparently dispensable for Ufd2p-mediated ubiquitination. In contrast, when K48 of the proximal ubiquitin was changed to arginine, only a single ubiquitin moiety could be transferred to Ub3-GFP regardless of whether the distal end ubiquitin included K48 (Fig. 2d, Lanes 8–10 and 18–20; Supplementary Fig. 5d). In this case, Ufd2p presumably only transfers ubiquitin to the middle ubiquitin moiety in Ub3-GFP. Likewise, when the K29R-WT-WT tri-ubiquitin was used in step I to assemble Ub4-GFP, Ufd2p transferred three ubiquitins onto Ub4-GFP, even when methylated ubiquitin was used (Fig. 2e, Lanes13–15; Supplementary Fig. 5e). These results further support the conclusion that Ufd2p preferentially adds ubiquitin conjugates to proximal ubiquitins in a K29-linked ubiquitin chain, except for the distal one. We next expressed and purified all the other HECT domain E3s in yeast and tested their activities in the Ub-V-GFP ubiquitination reaction. We found that Hul4p could not catalyse the ubiquitination of Ub-V-GFP. However, Rsp5p, Tom1p CT (a C-terminal truncation of Tom1p) and Hul5p could synthesize Ub2-GFP (Supplementary Fig. 6a–d). Among them, Hul5p synthesized both K29 and K48-linked Ub2-GFP (Supplementary Fig. 6e). Strikingly, Ufd2p could only add one ubiquitin to the K29-linked Ub2-GFP generated by Hul5p, but ignored the K48-linked Ub2-GFP that was present in the same reaction (Supplementary Fig. 6e). This observation further supports our conclusion that Ufd2p preferentially initiates branched chains from special linkages such as K29-linked ubiquitin conjugates.

It is noteworthy that, when we extended the reaction time to 2 h, we detected a small amount of high-molecular weight species of Ubn-GFP if WT Ub was used in step II. These high molecular weight species were reduced when either K48R Ub or Met-Ub was used in the second reaction (Fig. 2f, Lane 3 compared with Lanes 4 and 5). These results suggest that Ufd2p can form K48-linked ubiquitin chains, but only with low-efficiency. By contrast, the efficiency of Ufd2p-mediated multi-monoubiquitination was more readily detected (Fig. 2f; Supplementary Fig. 7a). Therefore, although we cannot completely exclude the possibility that Ufd2p might assemble some K48-linked polyubiquitin chains in cells, we still favour a model in which Ufd2p mainly synthesizes multi-monoubiquitin chains on K29-linked polyubiquitin chains. Based on these observations, we propose that Ufd2p is a chain branching enzyme rather than a chain elongation factor, it forms branched chains by preferentially adding a ubiquitin moiety to K48 non-distal ubiquitin molecules in a K29-linked oligo-ubiquitin chain synthesized by Ufd4p (Fig. 2g).

### *In vivo* branched ubiquitin chains assembly mediated by Ufd2p

**Figure 3:**
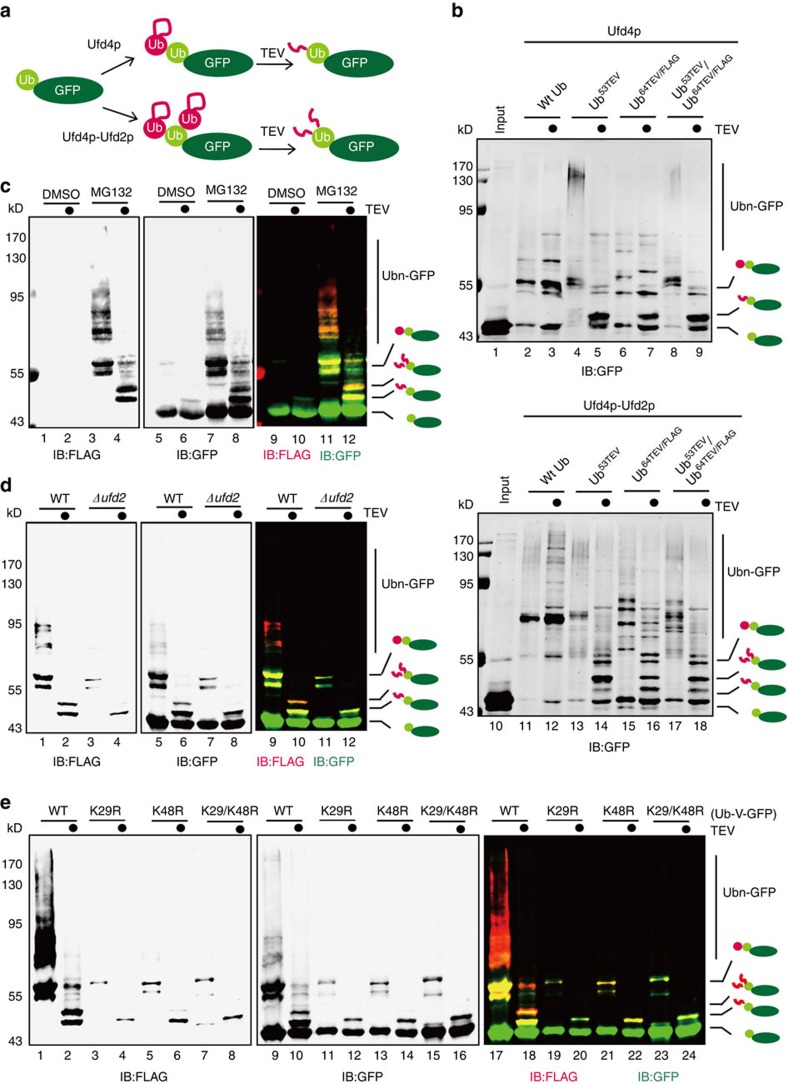
Branched ubiquitin chains are detected on UFD substrates in cells. (**a**) Schematic representation of the method used to monitor the synthesis of branched ubiquitin chains on Ub-V-GFP. (**b**) *In vitro* validation of the method used to monitor branched ubiquitin chains on Ub-V-GFP. As illustrated in (**a**), Ub-V-GFP was ubiquitinated by Ufd4p or Ufd4p-Ufd2p. Reaction products were treated with the TEV enzyme and analysed by immunoblotting with the anti-GFP antibody. (**c**) Ub-V-GFP was modified with branched ubiquitin chains in yeast cells. Ub-V-GFP and its modified forms were expressed in a wild-type strain expressing FLAG-Ub^53TEV^ and Myc-Ub^64TEV/FLAG^ and purified under denaturing conditions in the presence or absence of the proteasome inhibitor MG132. The pull-down products were treated with the TEV enzyme and analysed by immunoblotting with anti-FLAG and anti-GFP antibodies. (**d**) The addition of branched ubiquitin chains to Ub-V-GFP is mainly dependent on *UFD2 in vivo*. Ub-V-GFP and its modified forms were purified from either a wild-type or *ufd2Δ* strain expressing both FLAG-Ub^53TEV^ and Myc-Ub^64TEV/FLAG^ in the presence of MG132 under denaturing conditions. The pull-down products were treated with the TEV enzyme and analysed by immunoblotting with the anti-FLAG and anti-GFP antibodies. (**e**) K29 and K48 on Ub-V-GFP are required for the formation of branched ubiquitin chains in yeast cells. Wild-type, K29R, K48R or K29/48R Ub-V-GFP and their modified forms were purified from a wild-type strain expressing FLAG-Ub^53TEV^ and Myc-Ub^64TEV/FLAG^. The pull-down products were treated with the TEV enzyme and analysed by immunoblotting with the anti-FLAG and anti-GFP antibodies.

To determine whether Ufd2p and Ufd4p can form branched ubiquitin chains on Ub-V-GFP *in vivo*, we used a method developed by Meyer and Rape^14^. Briefly, a Tobacco Etch Virus protease (TEV) cleavage site was introduced into ubiquitin after Gly53, or after Glu64 in combination with a FLAG-epitope (hereafter referred to as Ub^53TEV^ and Ub^64TEV/FLAG^, respectively). Both ubiquitin variants could still be conjugated to Ub-V-GFP by Ufd4p and Ufd2p (Fig. 3b). After TEV digestion, a FLAG-epitope-containing peptide would remain attached to Ub-V-GFP if a single ubiquitin or a ubiquitin chain was conjugated to Ub-V-GFP. In contrast, if two ubiquitins were conjugated to Ub-V-GFP at the same time as in the case of branched chain formation, two FLAG peptide remnants would remain on Ub-V-GFP after TEV digestion (Fig. 3a). This method was first validated by Ufd4p and Ufd2p mediated *in vitro* ubiquitination: after TEV digestion, Ub-V-GFP containing one or two FLAG peptide remnants could be detected on Ub-V-GFP (Fig. 3b, Lanes 14, 16 and 18). If Ufd2p was omitted from the reaction, only cleaved Ub-V-GFPcontaining a single FLAG peptide was produced (Fig. 3b, Lanes 5, 7 and 9). These results suggested that this method is suitable for detecting branched ubiquitin chains on Ub-V-GFP.

We then co-expressed FLAG-Ub^53TEV^ and Myc-Ub^64TEV/FLAG^ with His-tagged Ub-V-GFP in yeast cells. Ub-V-GFP modified by these engineered ubiquitin variants was significantly enriched after treatment with the proteasome inhibitor MG132, suggesting that Ub-V-GFP modified by these ubiquitin variants is still targeted to the proteasome for degradation (Fig. 3c, Lanes 1, 5, 9 versus Lanes 3, 7, 11). We then purified Ub-V-GFP and the modified species from these cells under denaturing conditions using Ni-NTA agarose. After TEV digestion, Ub-V-GFP conjugated with two FLAG-containing peptides could be detected using either anti-FLAG and anti GFP antibodies (Fig. 3c, Lanes 4, 8 and 12), suggesting the presence of branched ubiquitin chains on Ub-V-GFP *in vivo*. In a *ufd2Δ* strain, ubiquitinated Ub-V-GFP was reduced compared with a wild-type strain, and the level of Ub-V-GFP conjugated with two FLAG-containing peptides decreased significantly. This suggested that the formation of branched ubiquitin chains on Ub-V-GFP is dependent on Ufd2p in yeast cells (Fig. 3d). In addition, we found that the presence of branched ubiquitin chains on Ub-V-GFP was dependent on both K29 and K48 of ubiquitin in the Ub-V-GFP molecule because mutating either of these residues to arginine almost abolished the production of branched ubiquitin chains on Ub-V-GFP (Fig. 3e). When Ub-K29R-V-GFP was used, Ub-V-GFP bearing only a single FLAG peptide could be detected, and when Ub-K48R-V-GFP was used, a small amount of Ub-V-GFP bearing two FLAG peptides was detected, which may resulted from the conjugation of a ubiquitin to a lysine in Ub-V-GFP other than K29 or K48. Taken together, these results suggest that Ufd2p can synthesize branched ubiquitin chains on Ub-V-GFP in yeast cells.

### Ufd2p binds K29-linked Ubn-GFP via its two N-terminal loops

**Figure 4:**
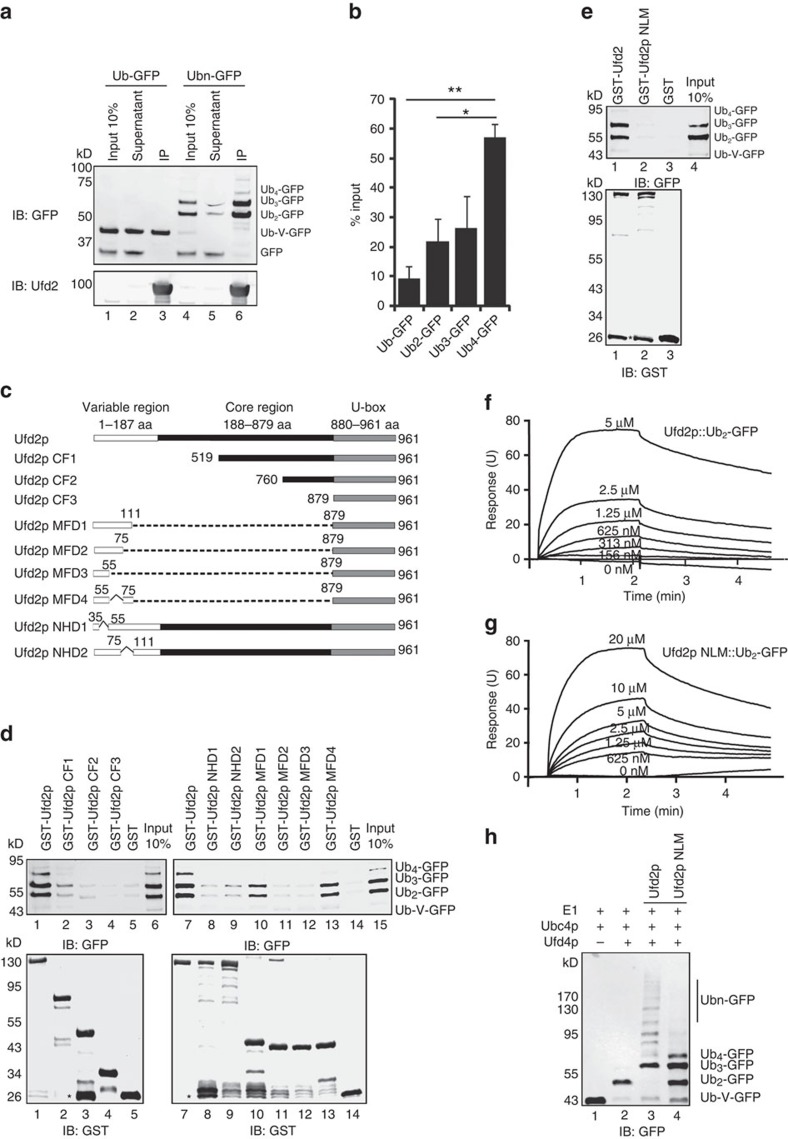
Ufd2p binds Ubn-GFP via two N-terminal loops. (**a**) Interactions between the substrate and Ufd2p. Biotinylated Ufd2p was immobilized on streptavidin-coated beads, which were then incubated with GFP, Ub-V-GFP or Ubn-GFP that was synthesized by Ufd4p. The precipitated proteins were analysed by immunoblotting with the indicated antibodies. (**b**) Quantification of the relative binding affinity between Ufd2p and Ubn-GFP. Relative amounts of Ub1-4-GFP were quantified using Odyssey software and compared with the input. Error bars denote the s.e.m. of three independent replicates. * indicated *P*<0.05, ^**^ indicated *P*<0.01. (**c**) A summary of the Ufd2p variants used to map the Ubn-GFP binding motif, which include Ufd2p (amino acids 1–961), Ufd2p CF1 (amino acids 519–961), Ufd2p CF2 (amino acids 760–961), Ufd2p CF3 (amino acids 879–961), Ufd2p MFD1(amino acids Δ111-879), Ufd2p MFD2(amino acids Δ75-879), Ufd2p MFD3(amino acids Δ55-879), Ufd2p MFD4(amino acids Δ55-75&111-879), Ufd2p NHD1(amino acids Δ35-55) and Ufd2p MFD2(amino acids Δ75-111). (**d**) Amino acids 35–55 and 75–111 of Ufd2p are necessary to bind to Ubn-GFP. GST-Ufd2p and its variants were purified and used to pull down Ubn-GFP, which was synthesized by Ufd4p; GST was used as a control. Asterisks in the bottom panel indicate GST products cleaved from the fused proteins. (**e**) Ufd2p binds to Ubn-GFP via its two N-terminal loops. GST-Ufd2p and GST-Ufd2p NLM (E51A K52A L53A D54A K55A E105A N106A M109A N110A) were purified and used to pull down Ubn-GFP. Asterisks in the bottom panel indicate GST products cleaved from GST-Ufd2p NLM. (**f**) SPR sensorgrams for the binding of GST-Ufd2p to K29-linked Ub2-GFP. A series of two-fold GST-Ufd2p dilutions was applied to the K29-linked Ub2-GFP surface (GFP served as a control). (**g**) SPR sensorgrams for the binding of GST-Ufd2p NLM to K29-linked Ub2-GFP. (**h**) The interaction between Ufd2p and Ubn-GFP is necessary for the E4 activity of Ufd2p. Ufd2p or Ufd2p NLM was incubated with E1, E2, Ufd4p, ATP, ubiquitin and Ub-V-GFP and detected using the anti-GFP antibody. See also Supplementary Figs 7–11.

Although Ufd2p itself could transfer ubiquitin to both Ub-V-GFP and Ubn-GFP (Fig. 2), the ubiquitination efficiency was much lower with Ub-V-GFP than with Ub2-V-GFP as the substrate (Supplementary Fig. 7a). These results suggested that Ufd2p prefers K29-linked ubiquitin chains as substrate. This substrate preference might arise from a specific interaction with substrates bearing a K29-linked ubiquitin chain. To test that possibility, we labelled Ufd2p with biotin and used the labelled proteins to pull down Ub-V-GFP and Ubn-GFP. We found that Ufd2p interacted with both Ub-V-GFP and Ubn-GFP (Fig. 4a, Lanes 3 and 6), but it bound substrates bearing a K29-linked chain with much higher affinity (Fig. 4a,b). According to surface plasmon resonance experiments, Ufd2p bound the K29-linked Ub2-GFP with a KD of 0.29 μM, whereas the KD was 2.03 μM for Ub-V-GFP (Fig. 4f and Table 1, Supplementary Fig. 7b and Supplementary Table 1). These results suggest an interaction between Ufd2p and the K29-linked ubiquitin chain.

Next, we defined the domain responsible for the higher affinity interaction of Ufd2p with substrates conjugated to K29-linked ubiquitin chains. To map this domain, we generated and purified a series of Ufd2p truncation mutants, as shown in Fig. 4c. GST pull-down experiments showed that deletion of the N-terminal region prevented Ufd2p from binding to Ubn-GFP (Fig. 4d, upper panel, Lanes 2–4; Supplementary Fig. 8a). Likewise, deleting either amino acids 55–879 or 75–879 of Ufd2p completely abolished its interaction with Ubn-GFP (Fig. 4d, upper panel, Lanes 11 and 12). In contrast, deleting the core region of Ufd2p (amino acids 111–879) did not affect the interaction with Ubn-GFP (Fig. 4d, upper panel, Lane 10). Thus, the substrate binding site is present in the N-terminal domain of Ufd2p.

The amino terminal region (amino acids 35–111) of Ufd2p contains three α-helices^30^. Further mapping experiments showed that while deleting the middle α-helix, 55-75aa, did not influence the interaction of Ufd2p with Ubn-GFP (Fig. 4d, Lane 13), deleting the 35-55aa or 75-111aa helices did reduce the interaction (Fig. 4d, Lanes 8 and 9). These results suggest that the Ubn-GFP binding activity of Ufd2p is dependent on these two regions. Further mutagenesis analyses (Supplementary Fig. 8c–f) showed that two loops in these regions (51-55aa and 105-110aa) were critical for substrate binding (Supplementary Fig. 8g); when residues in these two loops were mutated to alanine (Ufd2p NLM), the interaction between Ufd2p and Ubn-GFP was almost completely abolished (Fig. 4e). Surface plasmon resonance experiments confirmed that the affinity of Ufd2p NLM to K29-linked Ub2-GFP was lower than for WT Ufd2p, but there was no significant difference between Ufd2p or Ufd2p NLM binding to Ub-V-GFP (Fig. 4f–h, Supplementary Fig. 7b–c and Supplementary Table 1), suggesting that the two N-terminal loops of Ufd2p are involved in recognition of K29-linked Ub2-GFP. Importantly, this mutant also failed to form branched ubiquitin chains on Ufd4p synthesized Ubn-GFP (Fig. 4h, Supplementary Fig. 9a), although it had similar ubiquitin ligase activity as wild-type Ufd2p (Supplementary Fig. 9b). The defects of the NLM mutant in branched chain formation could not be attributed to either protein misfolding or reduced stability because: (1) Circular Dichroism (CD) spectral analysis showed that the secondary structure of Ufd2p NLM was similar to that of WT Ufd2p (Supplementary Fig. 10a); and (2) a cycloheximide chase experiment indicated that the degradation rate of Ufd2p NLM was similar to that of Ufd2p or Ufd2p ΔU-box (Supplementary Fig. 10b,c). Altogether, these results suggest that Ufd2p binds Ubn-GFP bearing a K29-linked ubiquitin chain via two N-terminal loops, which is critical for ubiquitin chain-branching activity.

Finally, we used the GST pull-down assay to test whether Ufd2p can recognize K48- and K63-linked di-ubiquitin. The result showed that Ufd2p did not interact with either K48- or K63-linked di-Ub (Supplementary Fig. 11a). The stepwise assay also showed that Ufd2p cannot extend ubiquitin chains on K48- or K63-linked Ubn-GFP (Supplementary Fig. 11b,c), which were synthesized by the Ube2g2-gp78 (ref. 25) (K48-linked Ubn-GFP) and Ubc13p/Mms2p (ref. 44) (K63-linked Ubn-GFP) ubiquitination systems, respectively. However, we found that Ufd2p could bind to Ub4-GFP that contains four ubiquitin moieties linked in a linear fashion (Supplementary Fig. 11d) and catalyse multi-monoubiquitination to this chain (Supplementary Fig. 11e). These results suggest that Ufd2p does not bind all ubiquitin linkages. Instead, it selectively binds to certain ubiquitin linkages, such as K29-linked and linear ubiquitin chains.

### Branched ubiquitin chains promote substrate degradation

**Figure 5:**
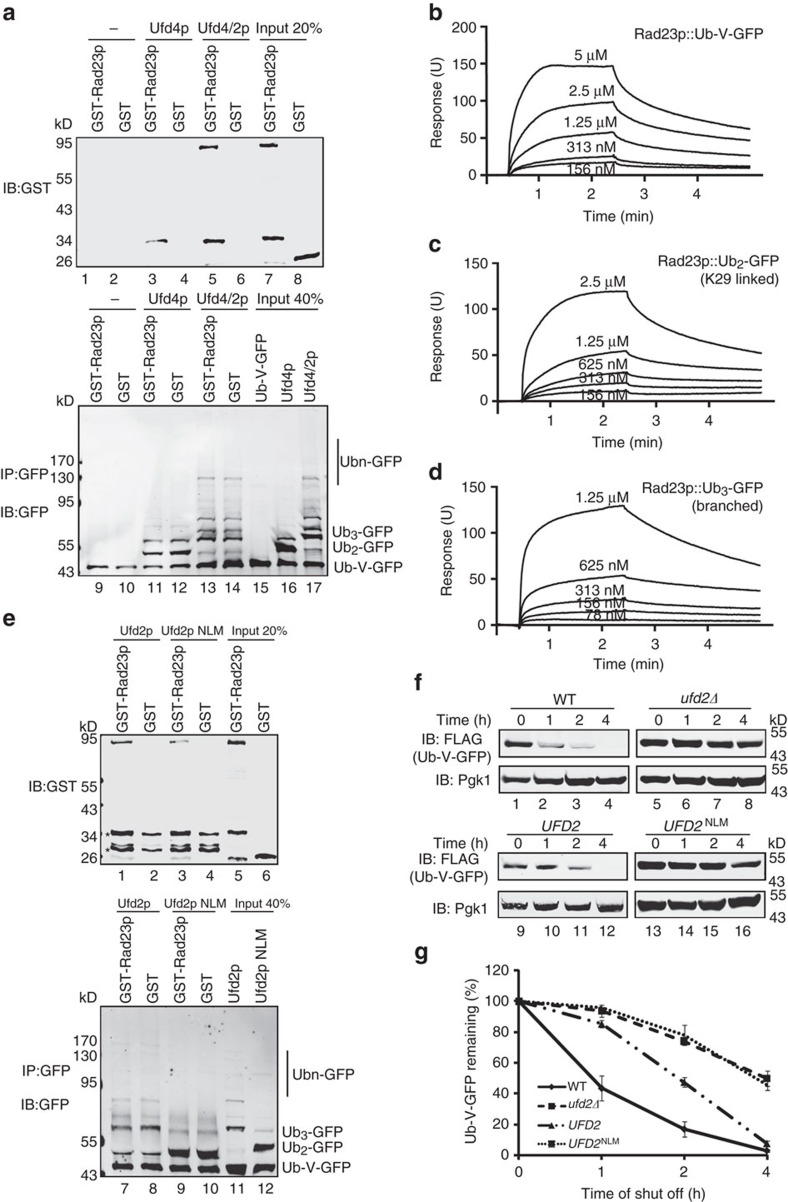
Substrates modified with branched ubiquitin chains are recognized by Rad23p, Dsk2p and Rpn10p. (**a**) Rad23p prefers to bind to branched ubiquitin chains on Ub-V-GFP over Ub-V-GFP with K29-linked ubiquitin chains or unmodified Ub-V-GFP. Ub-V-GFP, Ub-V-GFP with K29-linked ubiquitin chains (synthesized via Ufd4p) or Ub-V-GFP with branched ubiquitin chains (synthesized via Udf4p-Ufd2p) immobilized on protein A-sepharose beads were used to immunoprecipitate GST-Rad23p. The precipitated proteins were analysed by immunoblotting with the indicated antibodies. The asterisks indicate GST products cleaved from GST-Rad23p. (**b**) SPR sensorgrams for the binding of GST-Rad23p to Ub-V-GFP. A series of two-fold GST-Rad23p dilutions was applied to the Ub-V-GFP surface (GFP served as a control). (**c**) SPR sensorgrams for the binding of GST-Rad23p to K29-linked Ub2-GFP. (**d**) SPR sensorgrams for the binding of GST-Rad23p to branched Ub3-GFP. (**e**) Proteasome adaptor proteins such as Rad23p fail to recognize Ufd2p NLM-mediated ubiquitination products. Either wild-type Ufd2p-mediated ubiquitination products or Ufd2p NLM-mediated ubiquitination products immobilized on protein A-sepharose beads were used to immunoprecipitate GST-Rad23p. Ubn-GFP modified by Ufd2p NLM could not immunoprecipitate GST-Rad23p efficiently. Asterisks indicate nonspecific background signals from the IgG chains. (**f**) The ubiquitin chain linkage switch is necessary for substrate degradation. Protein expression in wild-type or *ufd2Δ* strains expressing Ub-V-GFP under the control of a galactose-induced promoter was stopped upon transfer to 2% glucose. The *ufd2Δ* strains harboured empty vector, *UFD2* or *UFD2*^NLM^ under the control of a galactose-induced promoter. The degradation of Ub-V-GFP over time was analysed by immunoblotting. Pgk1p served as a loading control. (**g**) Quantification of relative Ub-V-GFP levels in (**f**) using Odyssey software. Error bars denote the s.e.m. of three independent replicates. See also Supplementary Figs 12–14.

Next, we asked why Ufd2p is required for the degradation of a substrate modified by K29-linked ubiquitin chains. It is well established that K11- and K48-linked ubiquitin chains can target substrates to the proteasome for degradation^1^,^45^, and substrates conjugated with branched ubiquitin chains containing only K48 linkage can also be recognized and escorted to the proteasome^14^. It has been reported that Rad23p, Dsk2p and Rpn10p serve as ubiquitin receptors for the proteasome^46^,^47^. Therefore, we created constructs expressing GST-tagged Rad23p, Dsk2p and Rpn10p. We expressed and purified these GST-tagged proteins from *E. coli*, and used them in a pull down study with Ub-V-GFP, Ub-V-GFP modified with K29-linked ubiquitin chains or a K29-modified Ub-V-GFP that also bears a K48-linked ubiquitin branch. The results showed that Ub-V-GFP modified with branched ubiquitin chains, had a higher affinity for Rad23p, Dsk2p, and Rpn10p than either unmodified Ub-V-GFP or Ub-V-GFP modified with a K29-linked ubiquitin chain (Fig. 5a; Supplementary Fig. 12a,c: compare Lane 5 with Lanes 1 and 3). Because the ubiquitin-like domain of Rad23p interacts with the N-terminal region of Ufd2p^35^, it is possible that Rad23p co-immunoprecipitates with Ubn-GFP through Ufd2p rather than through a direct interaction. To rule out this possibility, we created a Ufd2 mutant construct, Ufd2 N mut-1 that could not bind Rad23p^35^, but still had normal ubiquitin ligase activity (Supplementary Fig. 13a,b). Ub-V-GFP modified with branched ubiquitin chains synthesized by Ufd2p N mut-1 could still bind to Rad23p with similar affinity to Ubn-GFP synthesized by wild-type Ufd2p (Supplementary Fig. 13b), suggesting a direct interaction between Rad23p and a Ub-V-GFP bearing branched ubiquitin chains. To further confirm this point, we purified K29-linked Ub2-GFP and Ub3-GFP containing a branched ubiquitin chain that had both K29 and K48 linkages and examined the interaction between Rad23p and Ub-V-GFP, K29-linked Ub2-GFP and branched Ub3-GFP by surface plasmon resonance experiments. Our results showed that Rad23p bound branched Ub3-GFP with a KD of 0.24μM, which was much lower than the KDs of Rad23p binding to K29-linked Ub2-GFP or Ub-V-GFP (Fig. 5b–d and Table 2). Consistent with the observation that the Ufd2p NLM mutant failed to efficiently catalyse K48-linked multi-monoubiquitination on Ubn-GFP, Rad23p, Dsk2p and Rpn10p proteins failed to recognize the reaction products (Fig. 5e; Supplementary Fig. 12b,d). Taken together, these results strongly suggest that once a K29-linked ubiquitin chain is switched to a branched chains bearing K48 linkages created by Ufd2p, the substrate can be efficiently recognized by the proteasome receptors.

To further demonstrate that linkage switching via branched chain formation is necessary for proteasomal degradation, we examined the degradation of Ub-V-GFP in yeast. Deletion of *UFD2* significantly inhibited the degradation of Ub-V-GFP (Fig. 5f, Lanes 5–8; Fig. 5g). Reintroducing wild-type *UFD2*, but not *UFD2*^NLM^ into *ufd2* deficient cells restored the degradation of Ub-V-GFP (Fig. 5f, Lanes 9–12 and 13–16; Fig. 5g). Although the N-terminal loops were closed to the Rad23p-interacting motif, a GST pull-down experiment showed that Ufd2p NLM bound to Rad23p in a similar as the wild-type Ufd2p (Supplementary Fig. 14). Thus, the defect of *UFD2*^NLM^ in catalysing Ub-V-GFP degradation is not because of a failed interaction with Rad23p. We concluded that the branched ubiquitin chain forming activity of Ufd2p is essential for the UFD degradation pathway, acting as a ubiquitin chain linkage switcher to alter a non-optimized degradation signal to a signal preferred by the ubiquitin receptors of the proteasome.

### The function of branched chains in ERAD and OLE pathways

**Figure 6:**
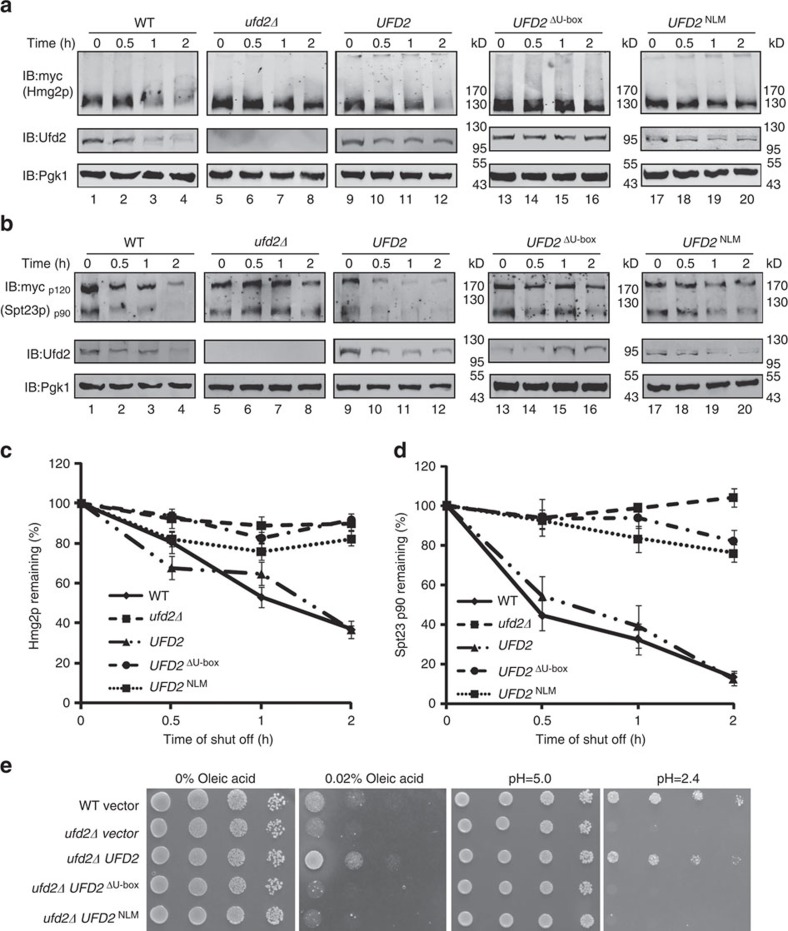
The branched chain forming activity of Ufd2p is required for ERAD, the OLE pathway and acid resistance in yeast. (**a**) The Ubn-GFP binding motif of Ufd2p is essential to the degradation of a subset of proteins during ERAD. Protein expression in wild-type and mutant strains (*ufd2Δ* strain contains empty vector, *UFD2*, *UFD2*^ΔU-box^ or *UFD2*^NLM^ under the control of a galactose-induced promoter) expressing Myc-Hmg2p under the control of a galactose-induced promoter was stopped by transfer to 2% glucose and addition of cycloheximide. The degradation of Myc-Hmg2p over time was analysed by immunoblotting. Pgk1p served as a loading control. (**b**) The Ubn-GFP binding motif of Ufd2p affects the stability of Spt23p in the OLE pathway. The degradation and detection of Myc-Spt23p expression under the control of a galactose-induced promoter were analysed as described above. (**c**) Quantification of Myc-Hmg2p turnover. The relative amounts of Myc-Hmg2p were quantified using Odyssey software; the high molecular weight smear representing the ubiquitylated species was considered when determining the turnover rate. Error bars denote the s.e.m. of three independent replicates. (**d**) Quantification of Myc-Spt23p p90 turnover. Error bars denote the s.e.m. of three independent replicates. (**e**) The Ubn-GFP binding motif of Ufd2p is essential to the OLE pathway and acidic resistance: 10-fold serial dilutions of the indicated strains (the wild-type strain contains empty vector; *ufd2Δ* strains contain empty vector, *UFD2*, *UFD2*^ΔU-box^ or *UFD2*^NLM^ under the *UFD2* promoter) were plated on media supplemented with either 0.02% oleic acid or 100 mM MES (pH 1.0) and grown at 30 °C for 2 days.

Ufd2p is known to be involved in ERAD (refs 32, 34) and in degradation of the short-lived HMG-CoA reductase, Hmg2p (refs 32, 48). Indeed, we observed strong stabilization of Hmg2p in the *ufd2Δ* strain (Fig. 6a,c). Wild-type *UFD2* fully restored the degradation of Hmg2p, but neither the *UFD2*^ΔU-box^ nor the *UFD2*^NLM^ mutant could do so (Fig. 6a,c). These results suggest that ubiquitin chain branching is essential for degradation of a physiological substrate in yeast.

Ufd2p also plays an important role in the OLE pathway in budding yeast by regulating the stability of Spt23p, a transcription factor that controls the synthesis of unsaturated fatty acids^37^. We therefore examined the degradation of Spt23p in a *UFD2*-deletion strain and found that the Spt23 p90 protein was significantly stabilized. Wild-type *UFD2*, but not the *UFD2*^ΔU-box^ or the *UFD2*^NLM^ mutant could fully restore Spt23p degradation in the *ufd2Δ* strain (Fig. 6b,d). Because one of the key target genes of Spt23p is *OLE1*, which regulates the synthesis of unsaturated fatty acids such as oleic acid, deregulation of this pathway is toxic to cells^49^,^50^. As a result, *ufd2Δ* mutants are sensitive to oleic acid^32^. Over-expression of wild-type *UFD2* made the *ufd2Δ* mutant less sensitive to oleic acid, but both *UFD2*^ΔU-box^ and *UFD2*^NLM^ failed to do so (Fig. 6e). In addition, we found that these two mutants also failed to rescue the growth defect of the *Δufd2* mutant under an acidic condition (Fig. 6e). Altogether, these results suggest that the N-terminal loops of Ufd2p are important in regulation of ERAD, the OLE pathway and cell growth under acidic conditions.

## Discussion

The diversity of ubiquitin code information is achieved via distinct forms of ubiquitination, such as monoubiquitination, multi-monoubiquitination and polyubiquitination via different linkages^2^,^8^,^10^,^11^,^12^. However, these ubiquitin signals are not necessarily static and can undergo further modification to alter the fate of substrate. For example, ubiquitin chains attached to a substrate can be removed by a deubiquitinase and revert to the fate conferred by the original ubiquitin modification^51^. In addition, a ubiquitin conjugate can be re-modelled or ‘edited' to execute a distinct function. Ubiquitin chain editing occurs when one type of chain is replaced by another^8^. A20, which contains an N-terminal deubiquitinating Ovarian Tumour (OTU) domain and a C-terminal RING domain, was proposed to be a chain-editing factor. A20 down-regulates NF-κB signalling through its OTU domain-mediated removal of K63-linked ubiquitin chains from the receptor interacting protein (RIP), and through the C-terminal RING domain catalysed polyubiquitination of RIP with K48-linked ubiquitin chains, which collectively results in proteasomal degradation of RIP (refs 52, 53, 54). The ubiquitin ligase RNF4 represents another type of conjugate modulator. Following modification of the substrate with a small ubiquitin-like modifier (SUMO), some SUMO-modified proteins can be ubiquitinated by RNF4 (refs 55, 56). Compared with A20, RNF4 is more effective in promoting substrate degradation, as it does not need to erase the original code. In this regard, branched ubiquitin chain formation by Ufd2p is in some ways analogous to SUMO dependent ubiquitination by RNF4. Although the exact function of K29-linked polyubiquitin chain is currently unknown, this type of ubiquitin conjugate does not appear to form an effective degradation signal as proteasome receptors such as Rad23p do not have high affinity to this ubiquitin conjugate^57^. Addition of additional ubiquitin conjugates in the form of branched ubiquitin chains by Ufd2p leads to more efficient degradation of the modified substrates by the proteasome.

Ufd2p was initially proposed to be a chain elongating factor because it can add ubiquitin conjugates to a substrate primed by another ligase^19^. However, the mechanism of Ufd2p-mediated ubiquitination had remained unclear. Previous studies have shown that Ufd2p can cooperate with an E2 enzyme in a manner similar to a RING ubiquitin ligase^30^, but the question remained as to how it collaborates with Ufd4p to assemble a productive degradation signal on diverse substrates. We previously proposed two possible models for Ufd2p- and Ufd4p-mediated ubiquitination: the sequential model and the cooperative model^30^. Here, we established a stepwise ubiquitination assay to distinguish between these two possibilities. We found that Ufd2p can independently add ubiquitin moieties to a UFD substrate as long as it carries a K29-linked ubiquitin chain formed by Ufd4p (Fig. 2b). We further demonstrate that Ufd2p preferentially added ubiquitin conjugates to proximal ubiquitin moieties on Ufd4p-synthesized K29-linked ubiquitin chains, resulting in branched ubiquitin chains on the substrate (Fig. 2c–g). Thus, Ufd2p and Ufd4p act in sequence and Ufd2p is a chain branching factor rather than a chain elongator.

Mechanistically, we found that Ufd2p prefers substrates bearing a K29-linked ubiquitin chain. The nature of substrate preference lies in a high affinity interaction between Ufd2p and Ubn-GFP bearing a K29-linked ubiquitin chain. Our mapping experiments show that the two loops in the N-terminal region of Ufd2p contribute to the substrate binding site, which is essential for the chain branching activity of Ufd2p. The crystal structure of Ufd2p has an elongated shape resembling a running dog: the E2 binding U-box domain forms the head and multiple irregular Armadillo-like repeats with two pronounced helical hairpin protrusions form the trunk^30^. The newly discovered substrate binding site is located in the tail, far from the U-box, raising the question of how Ufd2p transfers ubiquitin from a cognate E2 to the substrate. One possible solution is the use of a Ufd2p dimer in the chain branching reaction. In this case, the N-terminal motif recognizes K29-linked ubiquitin chains on a substrate and the U-box domain of a second Ufd2p catalyses the chain branching reaction. However, we have not found any evidence that supports oligomerization or dimerization of Ufd2p. Importantly, a mixture of the ΔU-box and Ufd2p NLM mutants could not restore Ufd2p activity (Supplementary Fig. 15), thus excluding the possibility that Ufd2p functions as a dimer. Alternatively, Ufd2p may catalyse ubiquitination in a manner analogous to the Skp1–Cul1–F-box-protein (SCF) ubiquitin E3 ligase complex, which shares a similar substrate and E2 binding configuration (Supplementary Fig. 16). The Skp1–Cul1–F-box-protein (SCF) ubiquitin E3 ligase complex contains a cullin subunit as a molecular scaffold^20^,^58^: its C-terminus interacts with a RING-finger protein, and the N-terminus interacts with the crucial substrate binding adaptor Skp1 (S-phase-kinase-associated protein-1), which in turn binds the F-box-protein and substrate^59^,^60^. Although the substrate-binding F-box protein is distant from the RING domain^61^, ubiquitin charged on E2 can be efficiently transferred to the substrate. This is thought to be achieved through conformational rearrangements modulated by a post-translational modification of the complex^62^,^63^. In this regard, Ufd2p may similarly undergo a conformational chain during the reaction to allow efficient transfer of ubiquitin to substrate.

**Figure 7:**
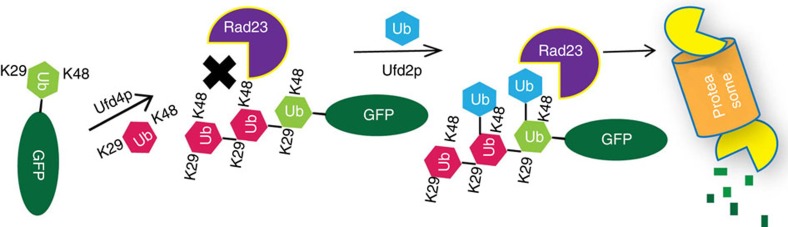
The functional role of Ufd2p in ubiquitination. Model of how Ufd2p functions as a ubiquitin chain-linkage switcher to promote the degradation of proteins conjugated to non-canonically linked ubiquitin chains.

Although branched ubiquitin chains have been detected both *in vivo* and *in vitro*^15^,^64^,^65^,^66^, the function of branched ubiquitin chains is still poorly understood. The anaphase-promoting complex (APC/C) can synthesize branched ubiquitin chains that contain both K11- and K48-linkages, and chain branching appears to strongly enhance substrate recognition by the proteasome, thereby promoting the degradation of several cell-cycle regulators during early mitosis^14^. In this study, we found that branched ubiquitin chains containing both K29 and K48 linkages participate in ERAD, the OLE pathway and acid resistance. Recently, K29-linked heterotypic ubiquitination has also been reported in mammalian cells^67^. Although K29-linked ubiquitin chains were found to direct the disassembly of certain proteins or to target proteins for lysosomal degradation^68^,^69^,^70^, there is no evidence that it could target substrates to the proteasome for degradation. It appears that K29-linked ubiquitin chain does not form a robust degradation signal for the proteasome. However, after being modulated into branched chains containing a K48 linkage, the modified substrate is capable of being targeted to the proteasome for degradation. Whether it is the branched ubiquitin chain or simply the K48 linkage that improves substrate degradation probability remains to be elucidated. However, given that several proteasome adaptor proteins are known to selectively recognize K48 linked ubiquitin chains, we propose that Ufd2p works as a ubiquitin chain branching ‘switch', that transforms a non-degradable ubiquitin signal to a type that is favored by the proteasome (Fig. 7), a paradigm that may be applicable to other non-canonical ubiquitin linkages (K6, K27 and K33).

## Methods

### Antibodies and proteins

The FLAG (1:2,000, M20008L), GST (1:2,000, M20007L), Myc (1:1,000, M20002M) and mouse GFP (1:1,000, M20004M) antibodies were purchased from Abmart (Shanghai, China), and the Xpress (1:2,000, R910-25) antibody was purchased from Invitrogen (Carlsbad, CA). The GFP (1:1,000), Ufd2 (1:1,000), and Pgk1(1:5,000) polyclonal antibodies were generated in rabbits using the corresponding recombinant proteins as antigens. The E1 enzyme, FLAG-ubiquitin, K48-linked di-ubiquitin, K63-linked di-ubiquitin and methylated ubiquitin were purchased from Boston Biochem (Cambridge, MA).

### Strains and plasmids

All yeast strains and plasmids used in this study are described in Supplementary Tables 2 and 3.

### Protein purification

Ufd2p and its variants, Ubc4p, Ub-V-GFP and its variants, Ufd3p, Otu1p, Rad23p, Dsk2p and Rpn10p, Hul4p, Hul5p, Rsp5p and C-terminal truncation of Tom1p were expressed as N-terminal hexahistidine-tagged or Glutathione S-transferase (GST)-tagged fusion proteins in pET28a or pGEX-4t-1 vectors. Briefly, the plasmid was introduced into Rosetta (DE3) cells and grown in Terrific Broth at 37 °C to an optical density of 0.8. The temperature of the culture was then shifted to 16 °C, and cells were induced with 0.25 mM isopropyl-D-thiogalactoside (IPTG) for 16 h. Cells were harvested by centrifugation and resuspended in lysis buffer (20 mM Tris, pH 7.4/300 mM NaCl/10 mM imidazole/10% glycerol for hexahistidine-tagged fusion protein; 50 mM Tris, pH 7.4/300 mM NaCl/2 mM MgCl2/5% glycerol for GST-tagged fusion proteins.) with 1 mM PMSF. After lysis by sonication and high-speed centrifugation of lysate, the supernatant was incubated with Ni Sepharose 6 Fast Flow (GE Healthcare, Marlborough, MA) or Glutathione Sepharose 4B (GE Healthcare, Marlborough, MA) for 2 h at 4 °C. The resin was washed, and the protein was eluted using the lysis buffer supplemented with 250 mM imidazole or 10 mM glutathione. Ufd4p was expressed under the control of the galactose-induced promoter in the SF10 yeast strain and purified under native conditions using Ni Sepharose 6 Fast Flow (GE Healthcare, Marlborough, MA). Ub-V-GFP, Ufd4p, Rad23p, Dsk2p, Rpn10p, Ufd3p, Otu1p and Ufd2p and its variants were further fractionated using size exclusion chromatography on a Superdex 75 or Superdex 200 column, in 50 mM Tris–HCl (pH 8.0), 150 mM potassium chloride, 5% glycerol and 2 mM magnesium chloride. FLAG-Ub mutants were purified under denaturing conditions (8 M urea) according to the instructions provided by Qiagen (Hilden, Germany). Purified proteins were refolded in PBS and dialyzed in the aforementioned protein purification buffer.

### *In vitro* ubiquitination assay

The ubiquitination experiments were conducted as follows: E1 (60 nM), Ubc4p (200 nM), Ufd4p (300 nM), Ufd2p (200 nM) and Ub-V-GFP (400 nM) were incubated with FLAG-Ub (10 nM) at 30 °C in buffer containing 25 mM Tris–HCl (pH 7.4), 2 mM magnesium/ATP and 0.1 mM DTT. Ubiquitination of Ub-V-GFP was detected by immunoblotting with an anti-GFP antibody. The immunoblotting was performed using a fluorescent dye-labelled secondary antibody (Invitrogen, Carlsbad, CA) and the blots were scanned using an Odyssey infrared imager.

### Single round ubiquitin turnover assay

To monitor a single round of Ub transfer from Ubc4p to Ub or its variants by Ufd2p, Ubc4p (300 nM) was incubated with E1 (60 nM) and FLAG-Ub K29R (10 nM, as the donor) at 30 °C for 15 min in the reaction buffer (25 mM Tris–HCl pH 7.4, 2 mM magnesium ATP, 0.1 mM DTT) and treated with 50 mM EDTA and 10 mM NEM for 15 min at room temperature. The Ub variants (10 × the volume of FLAG-Ub K29R) and Ufd2p (300 nM) were added at 30 °C. The reaction was stopped by the addition of SDS loading buffer. The samples were analysed by immunoblotting with the anti-FLAG antibody.

### Deubiquitylation reactions

The deubiquitylation activity of GST-Otu1p was performed as described below. The Ufd4p-Ufd2p ubiquitination product was incubated with GST-Otu1p in 50 mM NaCl, 50 mM Tris–HCl (pH 8.0), 5 mM Mg(OAc)_2_, 5% glycerol and 5 mM DTT. Reactions were incubated overnight at room temperature. The reaction was stopped by the addition of SDS loading buffer. The samples were analysed by immunoblotting with the anti-GFP antibody.

### Stepwise reaction assay

E1 (60 nM), Ubc4p (200 nM), Ufd4p (300 nM) and Ub-V-GFP (400 nM) were incubated with FLAG-Ub (10 nM) at 30 °C in the reaction buffer and treated with 50 mM EDTA and 10 mM NEM for 15 min at room temperature. The reaction products were incubated with the GFP antibody immobilized on protein A-sepharose beads (GE Healthcare, Marlborough, MA) at 4 °C for 2 h. After washing the beads three times with a low-salt buffer (50 mM Tris–HCl pH 7.5, 50 mM sodium chloride, 0.1% Triton), E1 (60 nM), Ubc4p (200 nM), Ufd2p (200 nM) and FLAG-Ub (10 nM) were added to the reaction buffer, and the reactions were incubated at 30 °C for 1 h. SDS loading buffer was added into the reactions, and the samples were analysed by immunoblotting.

### Synthesis of K29-linked ubiquitin oligomers

Ubiquitin oligomers carrying FLAG-UbK29R at the distal end were synthesized *in vitro* as described below. Briefly, E1(60 nM), Ubc4p (200 nM), Ufd4p (300 nM), Ub(D77) and FLAG-UbK29R (8 mg ml^−1^ each) were incubated in a buffer containing 25 mM Tris–HCl pH 7.4, 2 mM magnesium, 0.1 mM DTT and an ATP regenerating system at 30 °C for 1 h. At the end of the incubation, the reaction was treated with 1 mM DTT and 1 mM EDTA at 25 °C for 20 min. Yuh1 was added to the reaction at 20 μg ml^−1^. The reaction was further incubated at 30 °C for 1 h. Acetic acid (200 μl per reaction 2N) was added to the reaction to adjust the pH to ∼4. The acidified reaction mixture was applied to a Resource S column equilibrated with buffer A (50 mM ammonium acetate, pH 4.5, 1 mM EDTA, 5 mM DTT). The column was washed with three column volumes of buffer A, and the bound proteins were eluted with 10 column volumes of buffer A containing a linear gradient of sodium chloride (0–1.0 M). The fractions were collected and examined by immunoblotting with the FLAG antibody. This reaction yields mostly di-ubiquitin modifications, with FLAG-Ub K48R at the distal end. Tri-ubiquitin was synthesized using a similar method, except that various di-ubiquitin chains were used in the reaction.

### Branched chain detection

To detect branched linkages on Ub-V-GFP *in vitro*, the Ub-V-GFP ubiquitination experiment was conducted using Ufd4p and Ufd4p-Ufd2p as described above. FLAG-Ub, FLAG-Ub^53TEV^ and FLAG-Ub^64TEV/FLAG^ were used in these ubiquitination experiments. The TEV enzyme was added in excess, and the reactions were incubated for 16 h at 30 °C. Reactions were resolved via 12% Tricine SDS–polyacrylamide gel electrophoresis (SDS–PAGE).

To detect branched linkages on Ub-V-GFP *in vivo*, yeast strains harbouring either the pYEP-Ub-V-GFP-6 × His plasmid or its variants and the pESC-FLAG-Ub^53TEV^-Myc-Ub^64TEV/FLAG^ plasmid were grown in synthetic complete glucose medium (s.d., containing 0.67% (wt/vol) yeast nitrogen base supplemented with amino acids and 2% (wt/vol) dextrose) lacking uracil and leucine to an OD600 of 1.0. Yeast cells were harvested and washed three times with sterile water. The harvested cells were resuspended in synthetic complete galactose medium (containing 0.1 mM CuSO_4_) lacking uracil and leucine and grown for 16 h. Next, either DMSO or MG132 was added, and the cultures were incubated for 5 h. After treatment, the lysates were subjected to pull-down by using Ni-NTA agarose under denaturing conditions with denaturing buffer (10 mM Tris pH 8.0, 100 mM Na_2_HPO_4_/NaH_2_PO_4_ pH8.0, 6 M guanidine HCl, 10 mM NEM, 0.5% Triton). After exchanging the denaturing buffer for native buffer (20 mM Tris pH8.0, 300 mM NaCl, 10% Glycerol, 0.5% Tween 20, 5 mM EDTA, 10 mM NEM and 1 mM PMSF) via dialysis, the TEV enzyme was added in excess, and the reactions were incubated for 16 h at 30 °C. Reactions were resolved via 12% Tricine SDS–PAGE.

### Ufd2p binding assays

Biotin binding assays were performed as described below. Biotinylation labelling was performed using EZ-link sulfo-NHS-Biotin (Thermo Scientific, Waltham, MA) according to the manufacturer's instructions. The Ub-V-GFP and Ubn-GFP binding experiments were performed at 4 °C in 300 μl of a low-salt buffer (50 mM Tris, pH 7.4, 50 mM sodium chloride, 2.5 mM magnesium chloride, 0.1%TritonX-100 and 1 mg ml^−1^ BSA). Ub-V-GFP or Ubn-GFP was incubated with 5 μg of biotinylated Ufd2p immobilized on streptavidin beads. The precipitated complexes were analysed by immunoblotting, and the data were quantified using Odyssey software.

### GST pull-down experiments

GST-Ufd2p and its variants were incubated with Glutathione Sepharose 4B (GE Healthcare, Marlborough, MA) at 4 °C for 2 h. The GST protein was used as a negative control. The GST beads were centrifuged and washed with a high-salt buffer (50 mM Tris pH 7.4, 150 mM potassium chloride, 2 mM magnesium chloride, 0.1% Triton). Ufd4p-synthesized Ubn-GFP was added, and the beads were incubated at 4 °C for 2 h. The beads were centrifuged and washed three times with the high-salt buffer, and SDS loading buffer was added. The samples were analysed by immunoblotting with anti-GST and rabbit anti-GFP antibodies.

### Ubiquitin binding assays

Either Ub-V-GFP or Ubn-GFP, synthesized by Ufd4p or Ufd4p-Ufd2p, respectively, was coupled to protein A-sepharose beads (GE Healthcare, Marlborough, MA) with the immobilized anti-GFP antibody in a high-salt buffer at 4 °C for 2 h. After washing, GST-Rad23p, Dsk2p or Rpn10p were added, and the samples were incubated at 4 °C for 2 h. The beads were extensively washed three times and boiled in SDS loading buffer. The samples were analysed by immunoblotting with anti-GST or anti-GFP antibodies, respectively.

### Surface plasmon resonance (SPR) interaction analysis

Surface plasmon resonance interaction experiments were carried out at 25 °C on a Biacore 3,000 instrument (GE Healthcare, Marlborough, MA). GFP, Ub-V-GFP, K29-linked Ub2-GFP and branched Ub3-GFP were covalently attached to a carboxymethyl dextran-coated gold surface (CM5 Chip; GE Healthcare, Marlborough, MA) using an amide couple kit following the manufacturer's suggested protocol. Briefly, the carboxymethyl group of dextran was activated with N-ethyl-N′-(3-dimethylaminopropyl) carbodiimide (EDC) and *N*-hydroxysuccinimide (NHS). GFP, Ub-V-GFP, K29-linked Ub2-GFP and branched Ub3-GFP in a buffer containing 10 mM sodium acetate (pH 4.5) were injected and immobilized. Any remaining reactive sites in the flow cell were blocked with ethanolamine. Analysts were prepared in a buffer containing 10 mM HEPES (pH 7.4), 150 mM NaCl, 3 mM EDTA and 0.005% (vol/vol) surfactant P20. Regeneration was achieved by injecting a buffer containing 2.5 mM NaOH. The SPR signal was normalized using a reference flow cell containing GFP.

### Circular dichroism spectroscopy

To determine the secondary structure of GST-Ufd2p and GST-Ufd2p NLM, CD spectra of the proteins were collected at 0.1 mg ml^−1^ in 50 mM Tris (pH 8.0), 300 mM NaCl, 2 mM MgCl_2_, 10% Glycerol using a Chirascan Plus CD spectrometer (Applied Photophysics, Surrey, UK) and a 1 mm quartz sample cell equilibrated at 20 °C. Scans between 200 and 260 nm were performed at a scan rate of 50 nm per min.

### Expression shutoff experiments

For the Ub-V-GFP, Spt23p and Hmg2p degradation assays, yeast strains harbouring (1) the pESC-Ub-V-GFP-His6-FLAG plasmid and the pYC2-Ufd2 plasmid or its variants; (2) the pYes2-9 × myc Spt23 plasmid and the pESC-Ufd2 plasmid or its variants; or (3) the pYes2-4 × myc Hmg2 plasmid and the pESC-Ufd2 plasmid or its variants were grown in synthetic complete glucose medium (s.d., containing 0.67% (wt/vol) yeast nitrogen base supplemented with amino acids and 2% (wt/vol) dextrose) lacking uracil and leucine to an OD600 of 1.0. Yeast cells were harvested and washed three times with sterile water. The harvested cells were resuspended in synthetic complete galactose medium lacking uracil and leucine and incubated for 24 h. The expression of Ub-V-GFP, Myc-Spt23p or Myc-Hmg2p was shut off by changing the medium to 2% glucose. Translation was simultaneously stopped by adding cycloheximide to a final concentration of 1 mg ml^−1^. For each time point, yeast cells were harvested, and protein extracts were prepared as described below. The cells were resuspended in 100 ml distilled water, 100 ml 0.2 M NaOH was added, and the sample was incubated for 5 min at room temperature, pelleted, resuspended in 50 ml SDS sample buffer, boiled for 3 min and pelleted again. The Ub-V-GFP, Myc-Spt23p and Myc-Hmg2p levels were detected by immunoblotting analysis and quantified using Odyssey software. In the case of Hmg2p, the high molecular weight smear representing the ubiquitinated species was also considered to determine the protein turnover rate.

### Yeast growth sensitivity

Yeast strains were grown in synthetic complete medium lacking histidine (SD-His) at 30 °C to an OD600 of 1.0. The cultures were serially diluted 10 times, and each dilution was spotted onto SD-His plates containing either 0.02% oleic acid or 100 mM MES (pH=1.0). The plates were incubated at 30 °C for three days.

### Statistical analysis

All data are presented as the mean±s.e.m. The statistical significance of the differences between the mean values for the different genotypes was measured by Student's *t*-test with a paired, two-tailed distribution. The data were considered significant when the *P* value was less than 0.05 (*) or 0.01 (**)

### Data availability

The authors declare that all other data supporting the findings of this study are available within the article and its Supplementary Information files or from the corresponding author on reasonable request.

## Additional information

**How to cite this article:** Liu, C. *et al*. Ufd2p synthesizes branched ubiquitin chains to promote the degradation of substrates modified with atypical chains. *Nat. Commun.* **8,** 14274 doi: 10.1038/ncomms14274 (2017).

**Publisher's note:** Springer Nature remains neutral with regard to jurisdictional claims in published maps and institutional affiliations.

## Supplementary Material

Supplementary Information Supplementary Figures, Supplementary Tables and Supplementary References

## Figures and Tables

**Table 1:** SPR parameters of GST-Ufd2p and GST-Ufd2p NLM binding to K29-linked Ub2-GFP.

	**Ka (1/Ms)**	**Kd (1/s)**	**KA (1/M)**	**KD (μM)**
Ufd2p::Ub2-GFP	111	3.21e-5	3.46e6	0.29
Ufd2p NLM::Ub2-GFP	1.48e3	3.69e-3	4.01e5	2.49

**Table 2:** SPR parameters of GST-Rad23p binding to Ub-V-GFP, K29-linked Ub2-GFP and branched Ub3-GFP.

	**Ka (1/Ms)**	**Kd (1/s)**	**KA (1/M)**	**KD (μM)**
Rad23p::Ub-V-GFP	4.88e3	4.48e-3	1.09e6	0.92
Rad23p::Ub_2_-GFP	3.80e3	3.36e-3	1.01e6	1.00
Rad23p::Ub_3_-GFP	255	6.06e-5	4.22e6	0.24
