# Supplementary material for: Ufd2p synthesizes branched ubiquitin chains to promote the degradation of substrates modified with atypical chains
Source: Nat Commun. 2017 Feb 6;8:14274. doi: 10.1038/ncomms14274 (PMC5303827; doi:10.1038/ncomms14274)
Supplement: Supplementary Information — Supplementary Figures, Supplementary Tables and Supplementary References [file ncomms14274-s1.pdf]

Supplementary information

Supplementary Figure

Supplementary Figure 1

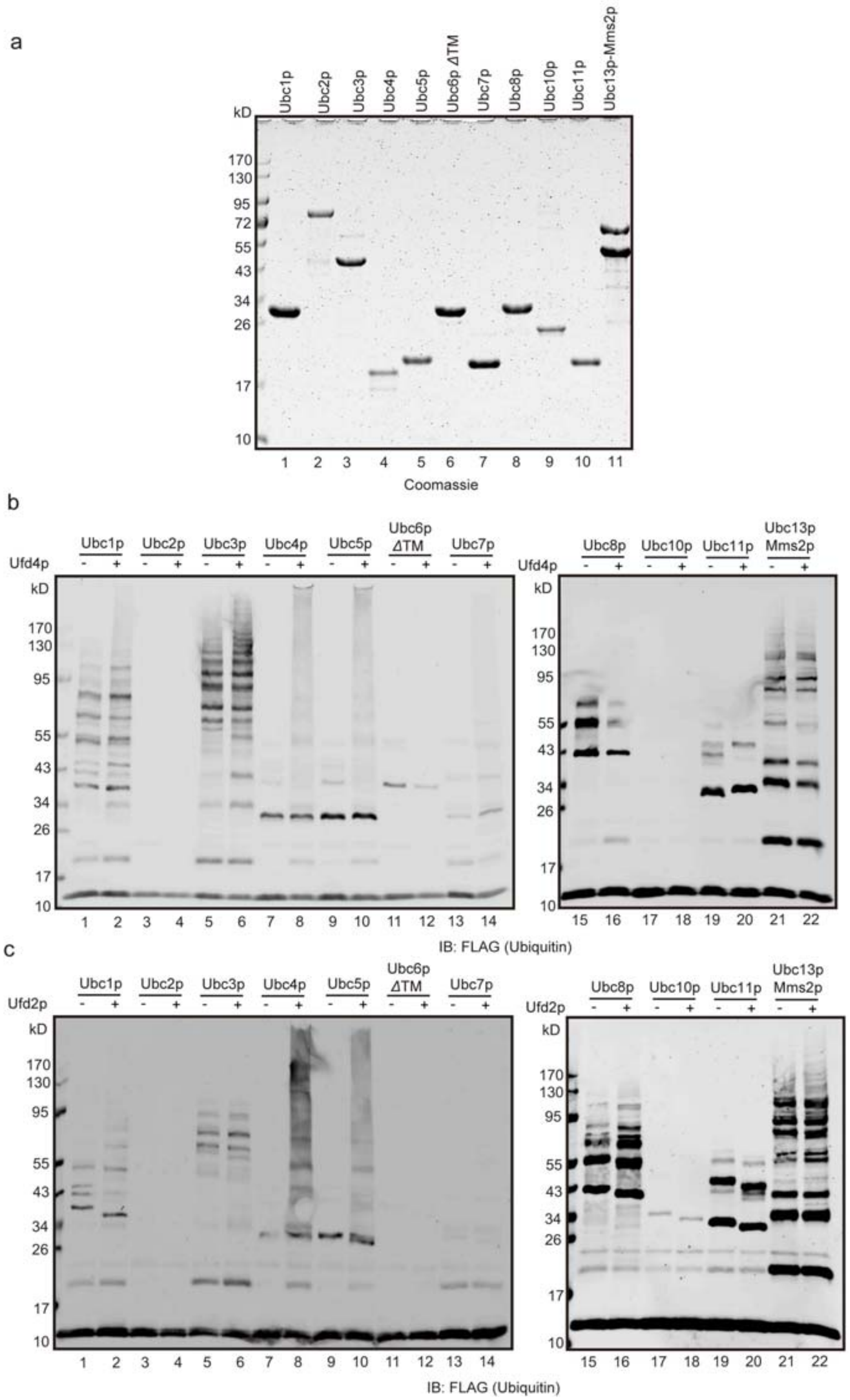

Supplementary Figure 1. Ubc4p is the appropriate E2 for Ufd4p or Ufd2p mediated ubiquitination, Relate to Figure 1.

(a) Coomassie blue-stained gels showing the expression and purification of relevant E2s of yeast.

(b) The E2s screening of Ufd4p-mediated *in vitro* ubiquitination system. E1, FLAG Ub, ATP and different E2s of yeast were incubated with or without Ufd4p at 30 degree for 30 min. The reaction products were detected by immunoblotting with anti-FLAG antibody.

(c) The E2s screening of Ufd2p-mediated *in vitro* ubiquitination system. E1, FLAG Ub, ATP and different E2s of yeast were incubated with or without Ufd2p at 30 degree for 30 min. The reaction products were detected by immunoblotting with anti-FLAG antibody.

Supplementary Figure 2

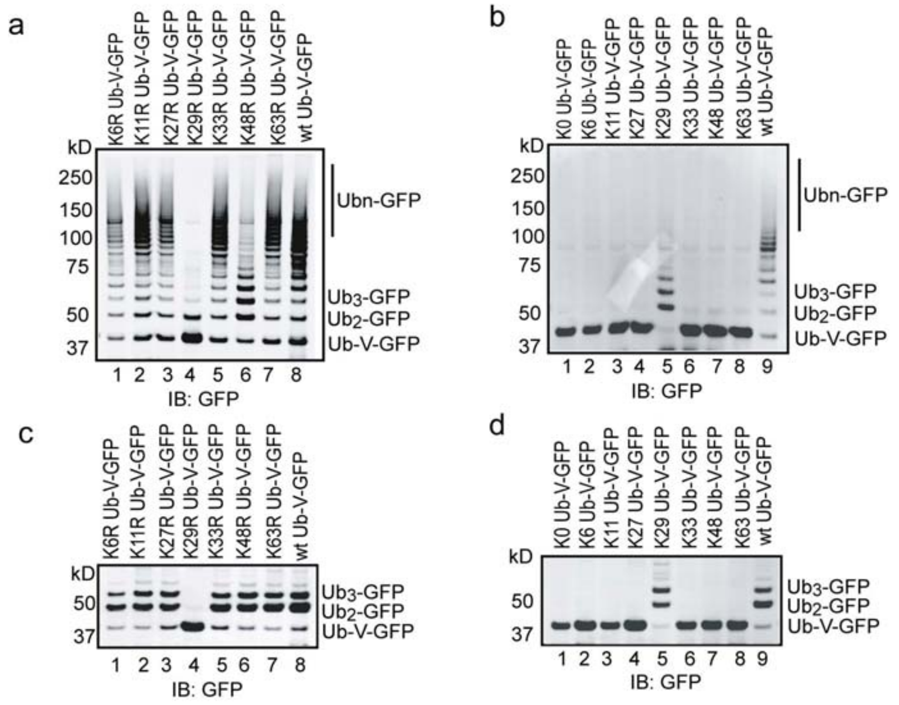

Supplementary Figure 2. Both Lys29 and Lys48 are necessary for Ufd4p-Ufd2p mediated polyubiquitination of Ub-V-GFP, Relate to Figure 1.

(a) K29R and K48R Ub-V-GFP are defective in Ufd4p-Ufd2p mediated polyubiquitination of Ub-V-GFP. Ub-V-GFP mutants, each containing one of its lysine residues changed to arginine in the ubiquitin region, were used as substrate in the Ufd4p-Ufd2p mediated ubiquitination. The reaction products were detected by immunoblotting with anti-GFP antibody.

(b) Neither K48 nor K29 only Ub-V-GFP could be fully polyubiquitinated by Ufd4p-Ufd2p mediated ubiquitination. Ub-V-GFP mutants, each containing only a single lysine in the ubiquitin region, were used as substrate in the Ufd4p-Ufd2p mediated ubiquitination.

(c) Lys29 is required for Ufd4p mediated ubiquitination. Just like (A), but Ufd2p was omitted in the reaction system.

(d) Lys29 is required for Ufd4p mediated ubiquitination. Just like in (B), but Ufd2p was omitted in the reaction system.

Supplementary Figure 3

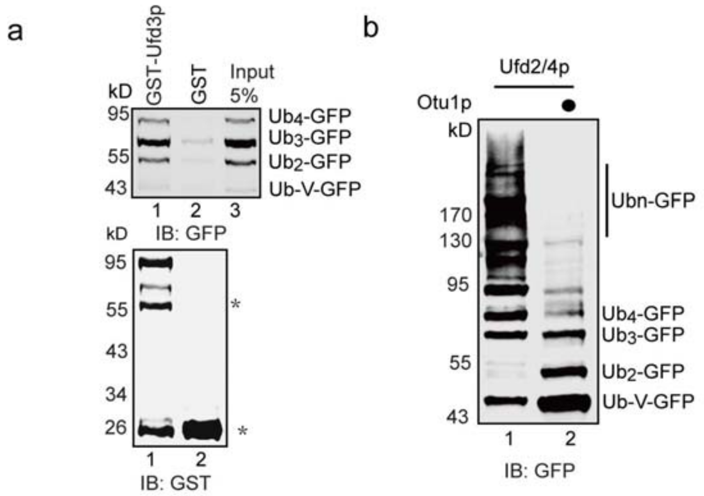

Supplementary Figure 3. Ubiquitin chain linkage-specificity of Ufd4p and Ufd2p mediated ubiquitination, Relate to Figure 1.

(a) GST-Ufd3p pull down Ufd4p synthesized Ubn-GFP. Purified GST-Ufd3p from *E. coli* was used to pull down Ubn-GFP, which was synthesized by Ufd4p. GST protein was used as control. Asterisks indicate GST products cleaved from the fused proteins.

(b) Otu1p removed the high molecular weight ubiquitination product of Ufd4p-Ufd2p reaction system. Ufd4p-Ufd2p synthesized Ubn-GFPs were incubated with Otu1p overnight at room temperature. Reactions were analyzed by immunoblotting with anti-GFP antibody.

Supplementary Figure 4

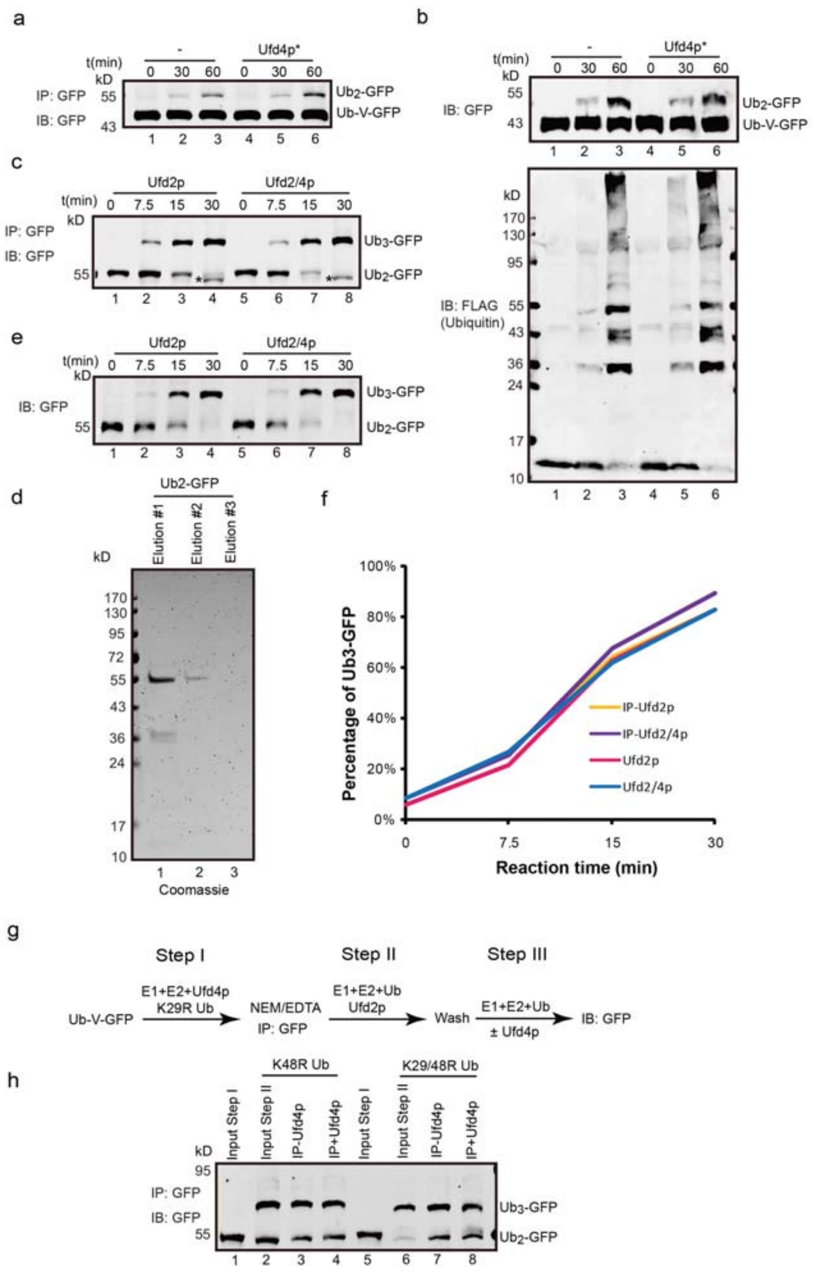

Supplementary Figure 4. The stepwise assay has the same ubiquitin transfer efficiency to Ufd4p-Ufd2p-mediated ubiquitination, Relate to Figure 2.

(a) The inactive Ufd4p did not influence Ufd2p-mediated Ub-V-GFP ubiquitination in the stepwise reaction assay. Ub-V-GFP in the absence or presence of Ufd4p treated with EDAT/NEM (Ufd4p*), then subjected to immunoprecipitate (IP) with the anti-GFP antibody. E1, E2, ubiquitin, ATP and Ufd2p were then added to the mixture. The reaction products were collected at the indicated times, and reaction products were detected by immunoblotting with anti-GFP antibody.

(b) The inactive Ufd4p did not influence Ufd2p's E3 ligase activity. Ufd4p was treated with EDAT/NEM, after dialyse off the NEM, the inactive Ufd4p (Ufd4p*) was added into the in vitro ubiquitination system, which contains E1, E2, Ufd2p, ubiquitin, ATP and Ub-V-GFP. Samples were collected at indicated times. The reaction products were detected by immunoblotting with anti-GFP antibody and anti-FLAG antibody.

(c) The active Ufd4p did not influence the efficiency of Ufd2p-mediated ubiquitination on Ub2-GFP in the stepwise reaction assay. Ub2-GFP which was synthesized by Ufd4p was subjected to immunoprecipitate (IP) with the anti-GFP antibody. Either Ufd2p or Ufd4p+Ufd2p together with E1, E2, K29R Ubiquitin and ATP were then added to the mixture. The reaction products were collected at the indicated times, and reaction products were detected by immunoblotting with anti-GFP antibody. Asterisks indicate Ufd2p-mediated ubiquitination products on Ub-GFP.

(d) The purification of Ufd4p synthesized Ub2-GFP. Ub2-GFP was synthesized by Ufd4p mediated ubiquitination system with FLAG-K29R Ub, and then purified by anti-FLAG M2 Affinity Gel (Sigma, A2220). The eluted fractions by FLAG peptides were dialyzed and detected with coomassie blue staining.

(e) The active Ufd4p did not enhance the efficiency of Ufd2p-mediated ubiquitination on Ub2-GFP. Either Ufd2p or Ufd4p+Ufd2p together with E1, E2, K29R Ubiquitin and ATP

was added to purified Ub2-GFP in (D). The reaction products were collected at the indicated times, and reaction products were detected by immunoblotting with anti-GFP antibody.

(f) Quantification of Ufd2p-mediated ubiquitination efficiency in (C) and (E). The percentage of Ub3-GFP in total Ub2- and Ub3-GFP was quantified by the Odyssey software

(g) Schematic representation of the stepwise ubiquitination experiment.

(h) Ufd4p could not extend from the Ub added by Ufd2 to produce highly branched structures. As illustrated in (g), Ub2-GFP synthesized by Ufd4p was subjected to immunoprecipitation (IP) with the anti-GFP antibody. E1, E2, K48R or K29/48R Ub, ATP and Ufd2p were then added to the mixture to produce branched Ub3-GFP. After extremely washing, E1, E2, K29R Ub, ATP and Ufd4p were added. The reaction products were detected using the anti-GFP antibody.

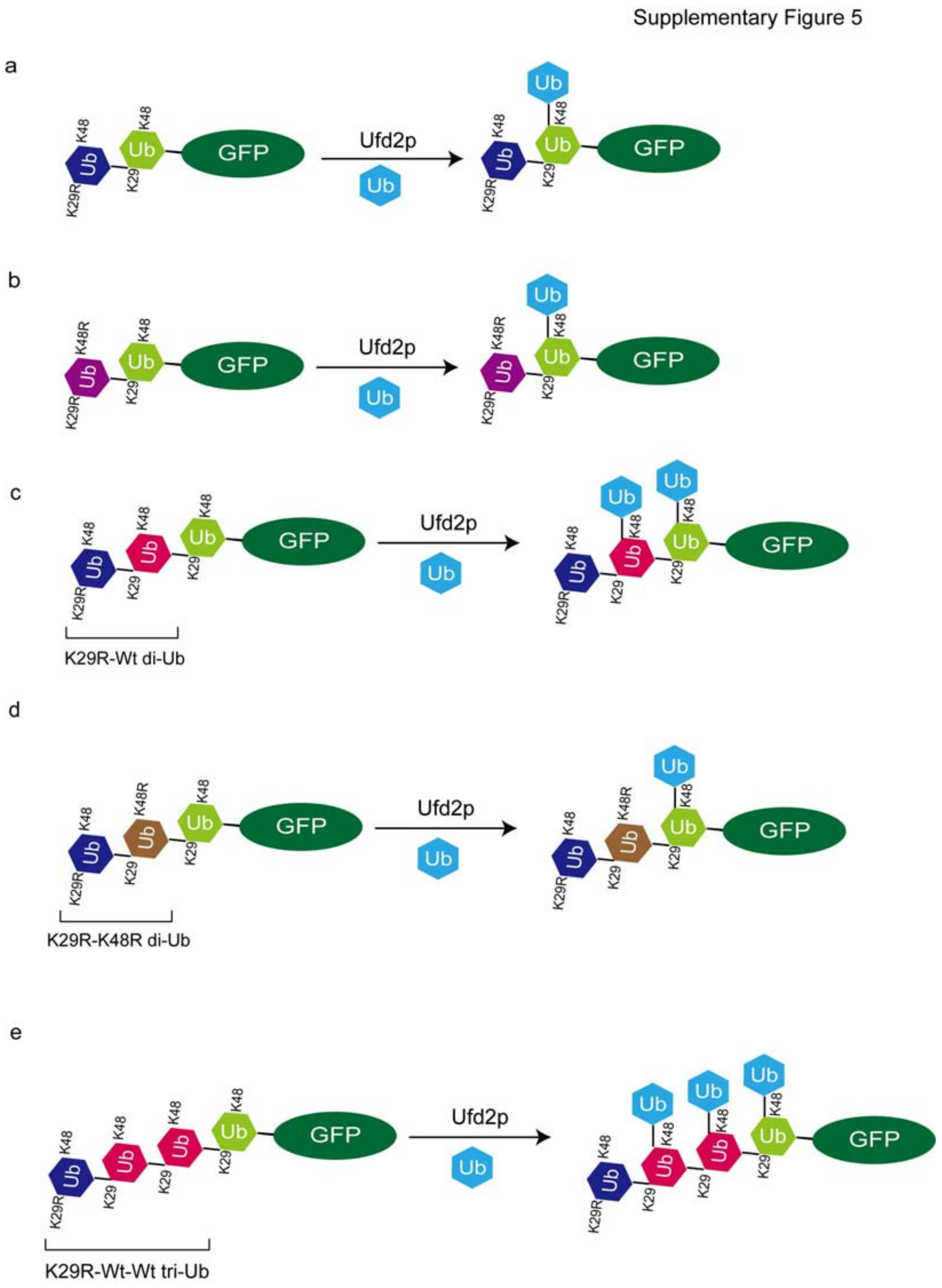

Supplementary Figure 5. Schematic representation of the topology of polyubiquitin chains on Ub-V-GFP, Relate to Figure 2.

(a) And (b) Schematic representation of the topology of Ufd4p-Ufd2p mediated polyubiquitination in Figure 2c.

(c) And (d) Schematic representation of the topology of Ufd4p-Ufd2p mediated polyubiquitination in Figure 2d.

(e) Schematic representation of the topology of Ufd4p-Ufd2p mediated polyubiquitination in Figure 2e (lanes 11-15).

Supplementary Figure 6

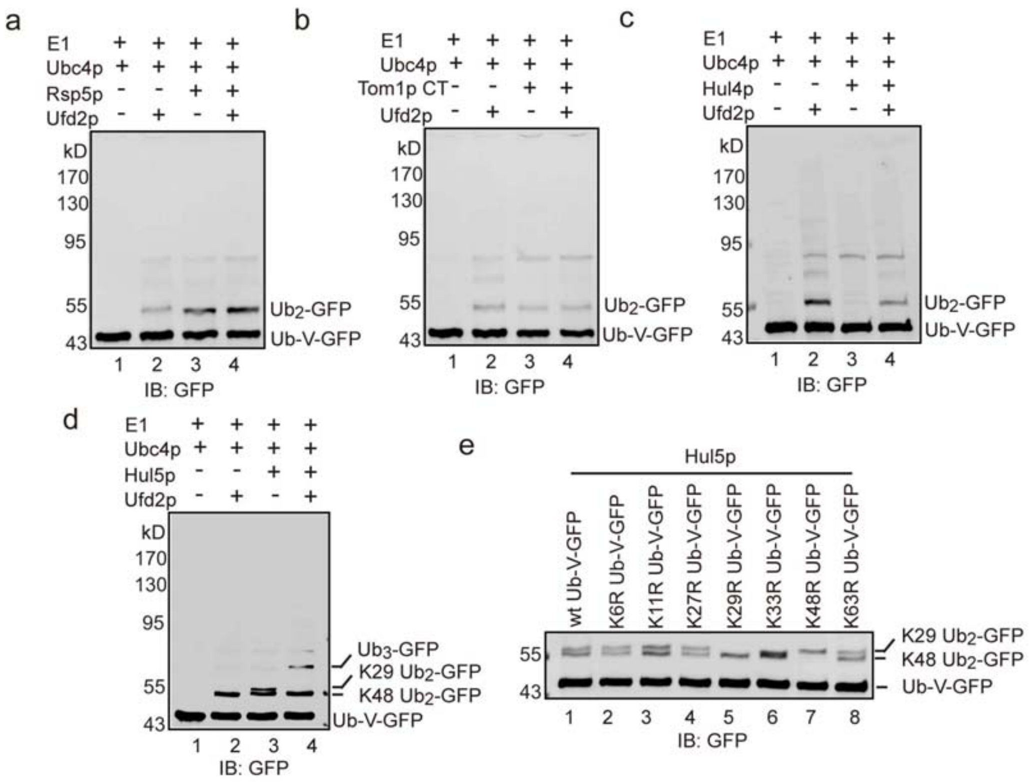

Supplementary Figure 6. HECT domain E3 ligases screening of the Ufd2p ubiquitination system, Relate to Figure 2.

(a) Ufd2p does not extend ubiquitin chains synthesized by Rsp5p. Ub-V-GFP ubiquitination was carried out *in vitro* using E1, E2, ubiquitin, ATP and Ub-V-GFP (lane 1). Where indicated, Ufd2p (lane 2), Rsp5p (lane 3), or both (lane 4) were added to the reaction..

(b) Ufd2p does not extend ubiquitin chains synthesized by Tom1p CT. Ub-V-GFP ubiquitination was carried out *in vitro* using E1, E2, ubiquitin, ATP and Ub-V-GFP (lane 1). Where indicated, Ufd2p (lane 2), Tom1p CT (lane 3), or both (lane 4) were added to the reaction.

(c) Hul4p does not extend ubiquitin chains on Ub-V-GFP. Ub-V-GFP ubiquitination was carried out *in vitro* using E1, E2, ubiquitin, ATP and Ub-V-GFP (lane 1). Where indicated, Ufd2p (lane 2), Hul4p (lane 3), or both (lane 4) were added to the reaction.

(d) Ufd2p can add one ubiquitin to Ub2-GFP synthesized by Hul5p. Ub-V-GFP ubiquitination was carried out *in vitro* using E1, E2, ubiquitin, ATP and Ub-V-GFP (lane 1). Where indicated, Ufd2p (lane 2), Hul5p (lane 3), or both (lane 4) were added to the reaction.

(e) Hul5p synthesized both Lys29 and Lys48-linked Ub2-GFP. Ub-V-GFP mutants, each with one of its lysine residues substituted to arginine, were used as the substrate in Hul5p-mediated ubiquitination. The reaction products were detected by immunoblotting with anti-GFP antibody.

Supplementary Figure 7

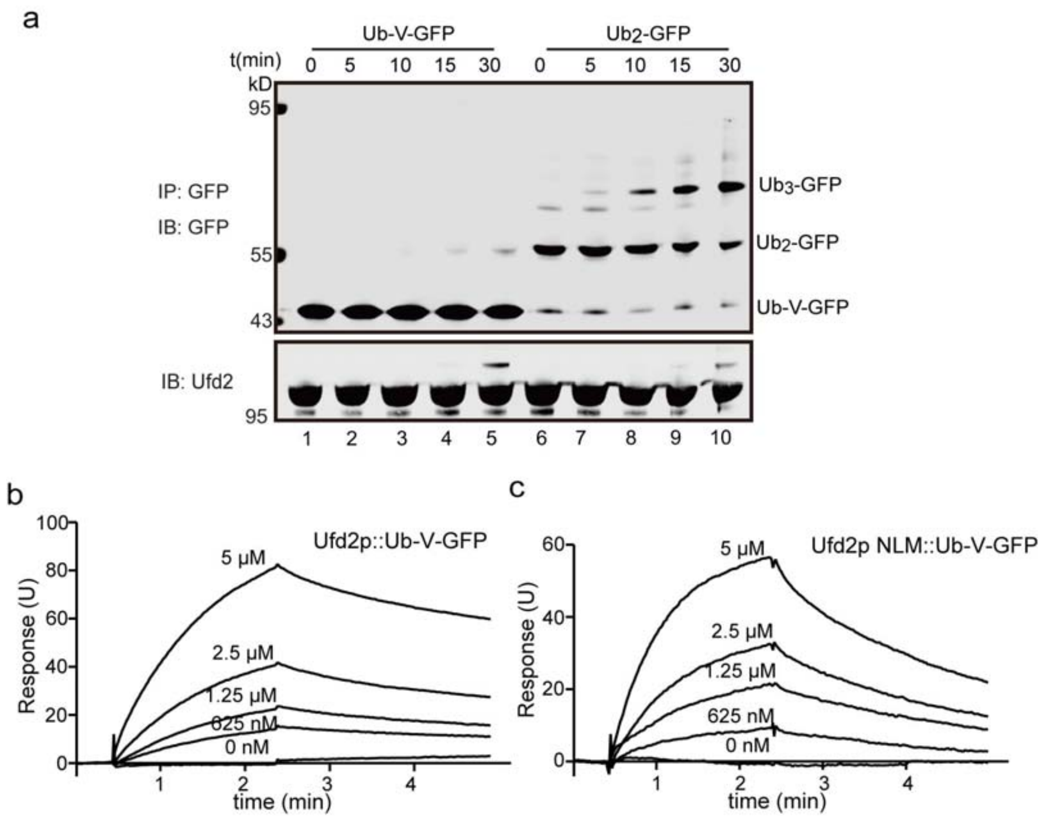

Supplementary Figure 7. The interaction between Ufd2p and Lys29-linked ubiquitin chain modified substrates might be essential to its physiological function, Relate to Figure 4.

(a) Ufd2p prefers to transfer ubiquitin to Ub2-GFP rather than Ub-V-GFP. Either Ub-V-GFP or Ub2-GFP which was synthesized by Ufd4p was subjected to immunoprecipitation (IP) with the anti-GFP antibody. E1, E2, ubiquitin, ATP and Ufd2p were then added to the mixture. The reaction products were collected at the indicated times, and reaction products were detected by immunoblotting with anti-GFP antibody.

(b) SPR sensorgrams for the binding of GST-Ufd2p to Ub-V-GFP. A series of 2-fold GST-Ufd2p dilutions was applied on Ub-V-GFP surface (GFP worked as control).

(c) SPR sensorgrams for the binding of GST-Ufd2p NLM to Ub-V-GFP.

Supplementary Figure 8

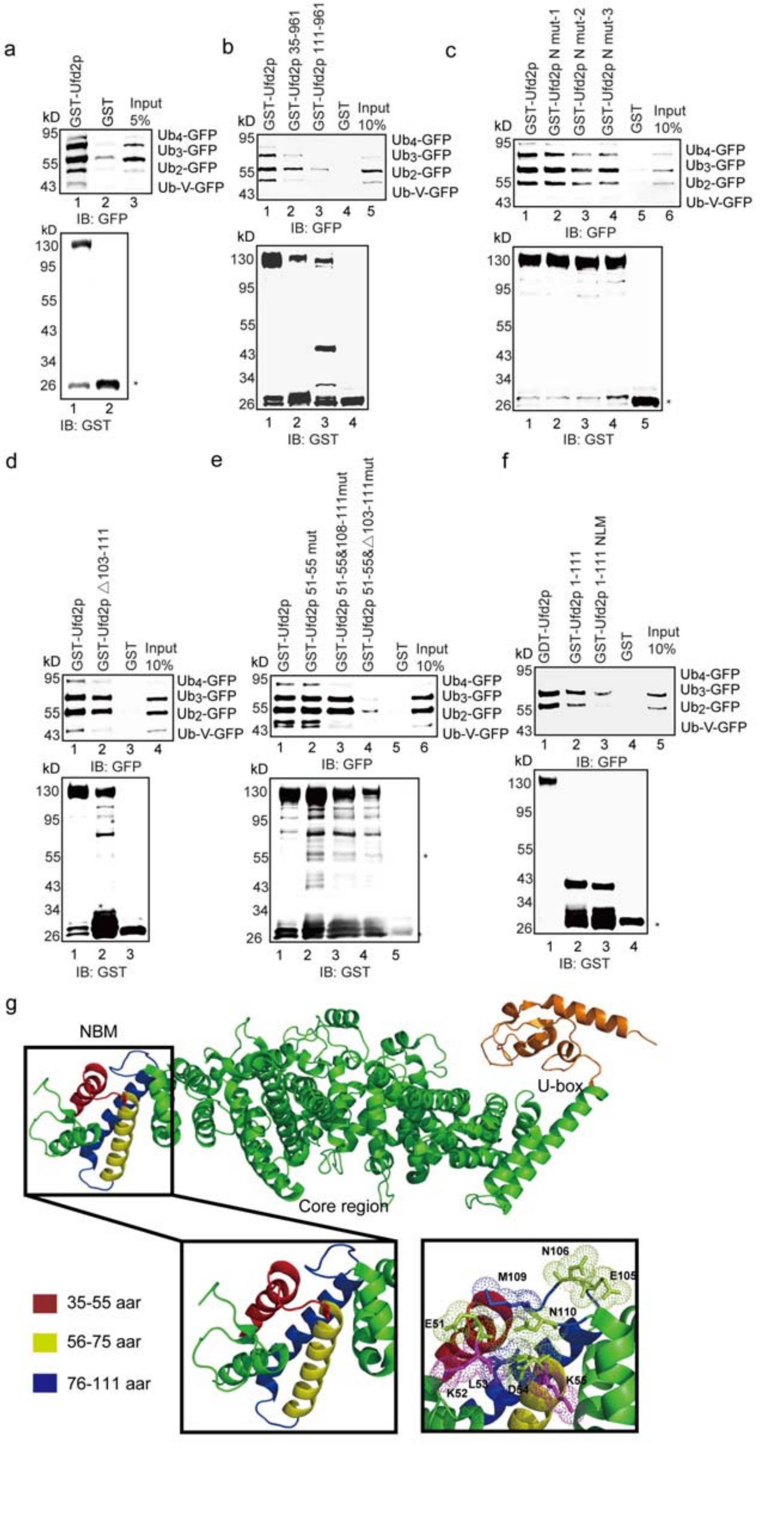

119 7

Supplementary Figure 8. Ufd2p binds Ubn-GFP by two N-terminal loops, Relate to Figure 4.

(a) GST-Ufd2p pull down Ufd4p synthesized Ubn-GFP. Purified GST-Ufd2p from *E. coli* was used to pull down Ubn-GFP, which was synthesized by Ufd4p. GST protein was used as a control group.

(b) Ufd2p 35-111aa is necessary to bind to Ubn-GFP. Purified GST-Ufd2p, Ufd2p 35-961aa and Ufd2p 111-961 aa were used to pull down Ubn-GFP, which was synthesized by Ufd4p. GST protein was used as a control group.

(c) Some Ufd2p N terminal mutant proteins did not affect their interaction with Ubn-GFP. GST-Ufd2, Ufd2p N mut-1(L44A Y97A V100A I104A F107A), Ufd2p N mut-2(L44A Y97A V100A F107A L93A G96A), Ufd2p N mut-3(L44A Y97A V100A I104A F107A L93A G96A) were used to pull down Ubn-GFP, which was synthesized by Ufd4p. GST protein was used as a control group.

(d)Ufd2p 103-111aa deletion mutant protein did not affect its interaction with Ubn-GFP. Purified GST-Ufd2p and Ufd2p Δ103-111aa were used to pull down Ubn-GFP, which was synthesized by Ufd4p. GST protein was used as a control group.

(e) Two N-terminal loops of Ufd2p are necessary to bind to Ubn-GFP. Purified GST-Ufd2p, Ufd2p 51-55mut(E51A K52A L53A D54A K55A), Ufd2p 51-55&108-111 mut (E51A K52A L53A D54A K55A M109A N110A), Ufd2p 51-55&Δ103-111 mut (E51A K52A L53A D54A K55A&Δ103-111aa) were used to pull down Ubn-GFP, which was synthesized by Ufd4p. GST protein was used as a control group.

(f) The N-terminal fragment of Ufd2p is sufficient for binding to Ubn-GFP. Purified GST-Ufd2p, Ufd2p 1-111aa and Ufd2p 1-111aa NLM were used to pull down Ubn-GFP, which was synthesized by Ufd4p. GST protein was used as a control group.

(g) The Ubn-GFP binding motif localizes to the N-terminal region of Ufd2p. In the structure of Ufd2p (2QIZ), green indicates the core region, orange indicates the U-box domain, and red (35-55aa), yellow (56-75aa) and blue (76-111aa) indicate the three α-helices in the N-

terminus of Ufd2p, respectively. The residues that might be involved in binding to Ubn-GFP are shown as sticks with electron clouds. NBM: N-terminal binding motif.

Supplementary Figure 9

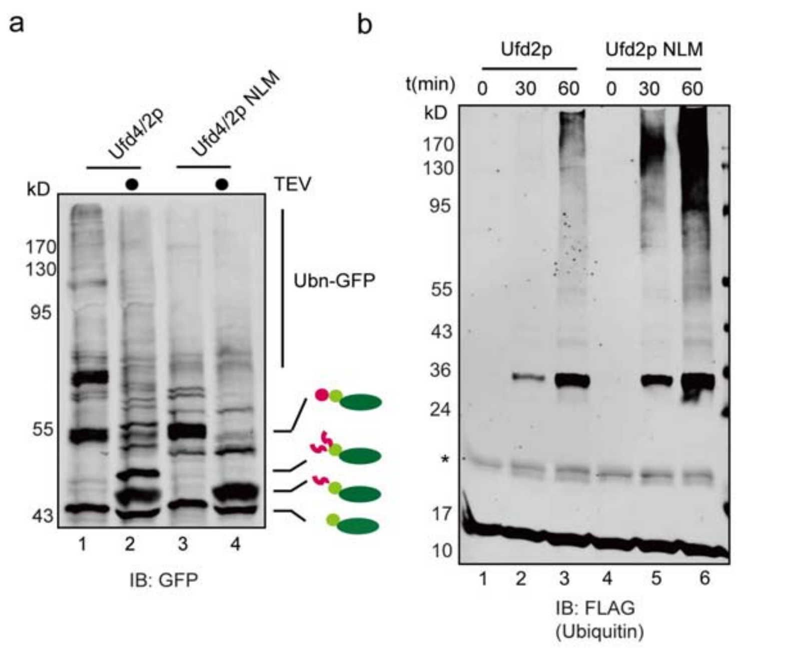

Supplementary Figure 9. Ufd2p NLM is deficient in assembly branched ubiquitin chains on Ub-V-GFP, but does not affect its E3 ligase activity, Relate to Figure 4.

(a) Ufd2p NLM failed to assembly branched ubiquitin chain on Ub-V-GFP. Ub-V-GFP was modified by Ufd4p-Ufd2p or Ufd4p-Ufd2p NLM by using FLAG-Ub^{53TEV}. Reaction products were treated with the TEV enzyme and analyzed by immunoblotting with the anti-GFP antibody.

(b) The E3 ligase activity of Ufd2p NLM was not impaired. Either Ufd2p (lanes 1-3) or Ufd2p NLM (lanes 4-6) were added to the ubiquitination system *in vitro*, which contains E1, E2, FLAG-Ub and ATP. The reaction products were collected at the indicated time pointes, and free ubiquitin chain which were synthesized by Ufd2p were detected by immunoblotting with the anti-FLAG antibody.

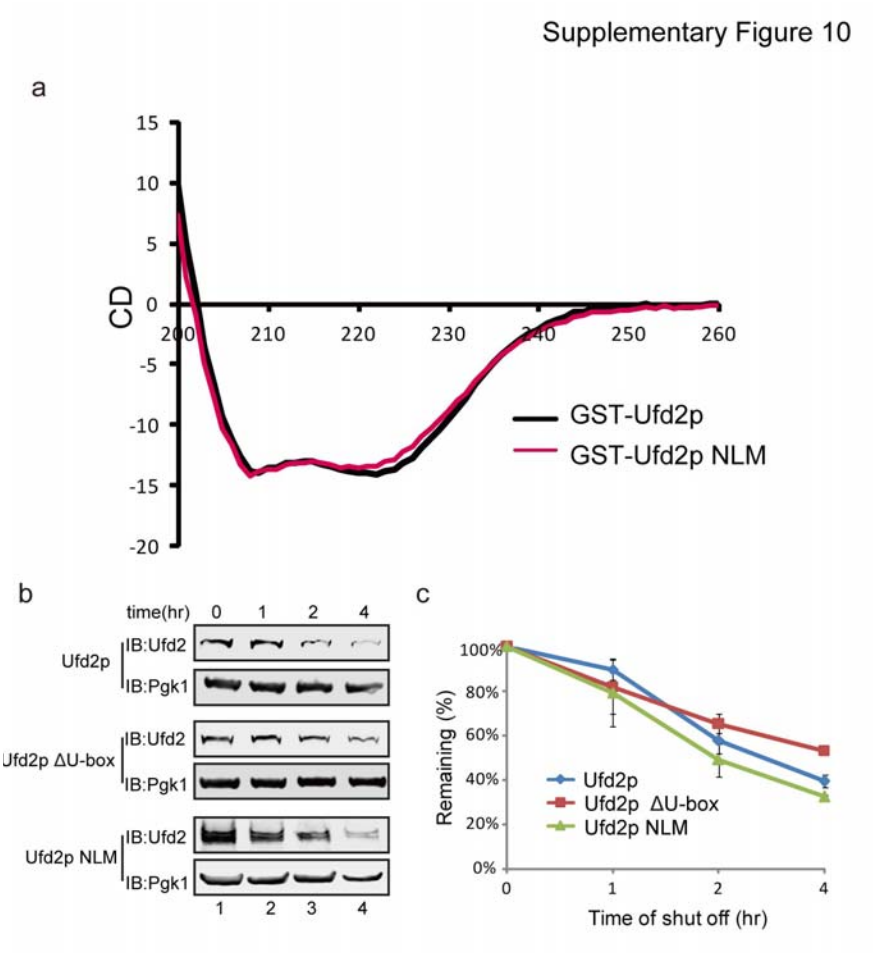

Supplementary Figure 10. The folding and stability of Ufd2p NLM are not affected, Relate to Figure 4.

(a) CD spectroscopy analyses of GST-Ufd2p and GST-Ufd2p NLM.

(b) The stability of Ufd2p NLM was not obviously influenced. Protein expression in *ufd2Δ* strains expressing Ufd2p, Ufd2p ΔU-box, and Ufd2p NLM under the control of a galactose-induced promoter was stopped upon transfer to 2% glucose. The degradation of Ufd2p, Ufd2p ΔU-box, and Ufd2p NLM over time was analyzed by immunoblotting. Pgk1p served as a loading control.

(c) Quantification of the relative Ufd2p, Ufd2p ΔU-box, and Ufd2p NLM levels in (b) by using the Odyssey software.

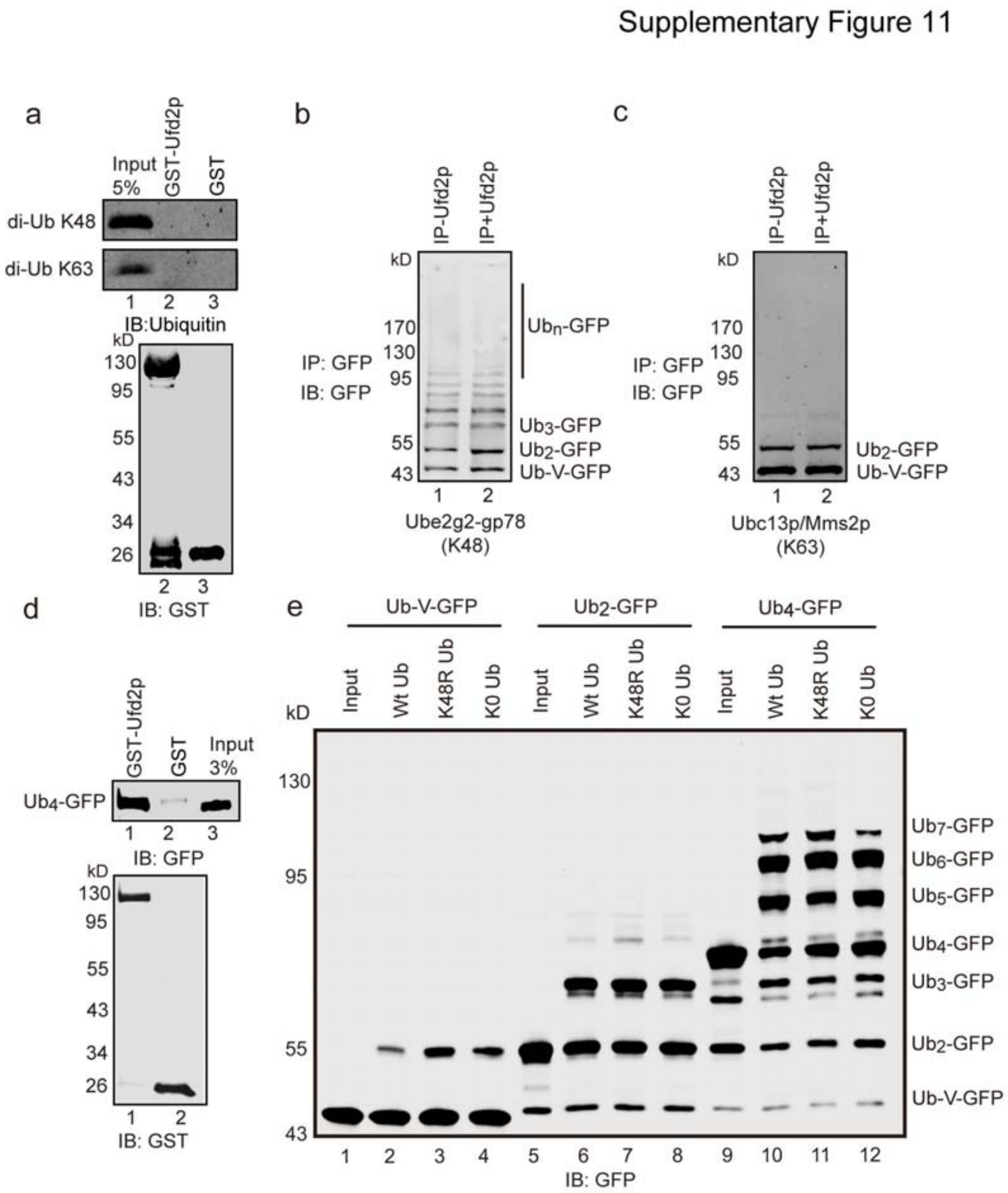

Supplementary Figure 11. Ufd2p could recognize linear ubiquitin chains but not Lys48 or Lys63-linked ubiquitin chains, Relate to Figure 4.

(a) Ufd2p does not bind with Lys48- or Lys63-linked di-Ub. Purified GST-Ufd2p was used to pull down Lys48 or Lys63-linked di-Ub. GST protein was used as a control.

(b) Ufd2p does not extend ubiquitin chains on Lys48-linked Ubn-GFP synthesized by Ube2g2-gp78. Lys48-linked Ubn-GFP synthesized by Ube2g2-gp78 was purified by protein A beads immunobilized with anti-GFP antibody. The beads were then incubated with E1, E2,

Ubiquitin, ATP in the absence (-) or presence (+) of Ufd2p. The reaction products were analyzed by immunoblotting with anti-GFP antibody.

(c) Ufd2p does not extend ubiquitin chains of Lys63-linked Ubn-GFP. Lys63-linked Ubn-GFP synthesized by Ubc13p/Mms2p was purified by protein A beads immunobilized with anti-GFP antibody. The beads were then incubated with E1, E2, Ubiquitin, ATP in the absence (-) or presence (+) of Ufd2p. The reaction products were detected by immunoblotting with anti-GFP antibody.

(d) Ufd2p interacts with Ub4-GFP. Purified GST-Ufd2p was used to pull down Ub4-GFP containing 4 ubiquitin moieties in tandem. GST protein was used as a control.

(e) Ub-V-GFP (lanes 2-4), Ub2-GFP (lanes 6-8) or Ub4-GFP (lanes 8-12) was added to the Ufd2p ubiquitination system *in vitro*, which contains E1, E2, ubiquitin (WT Ub, K48R Ub, K0 Ub), ATP and Ufd2p, and reaction products were analyzed by immunoblotting with anti-GFP antibodies. Lanes 1, 5, 7 show the purified Ub-V-GFP variants (input).

Supplementary Figure 12

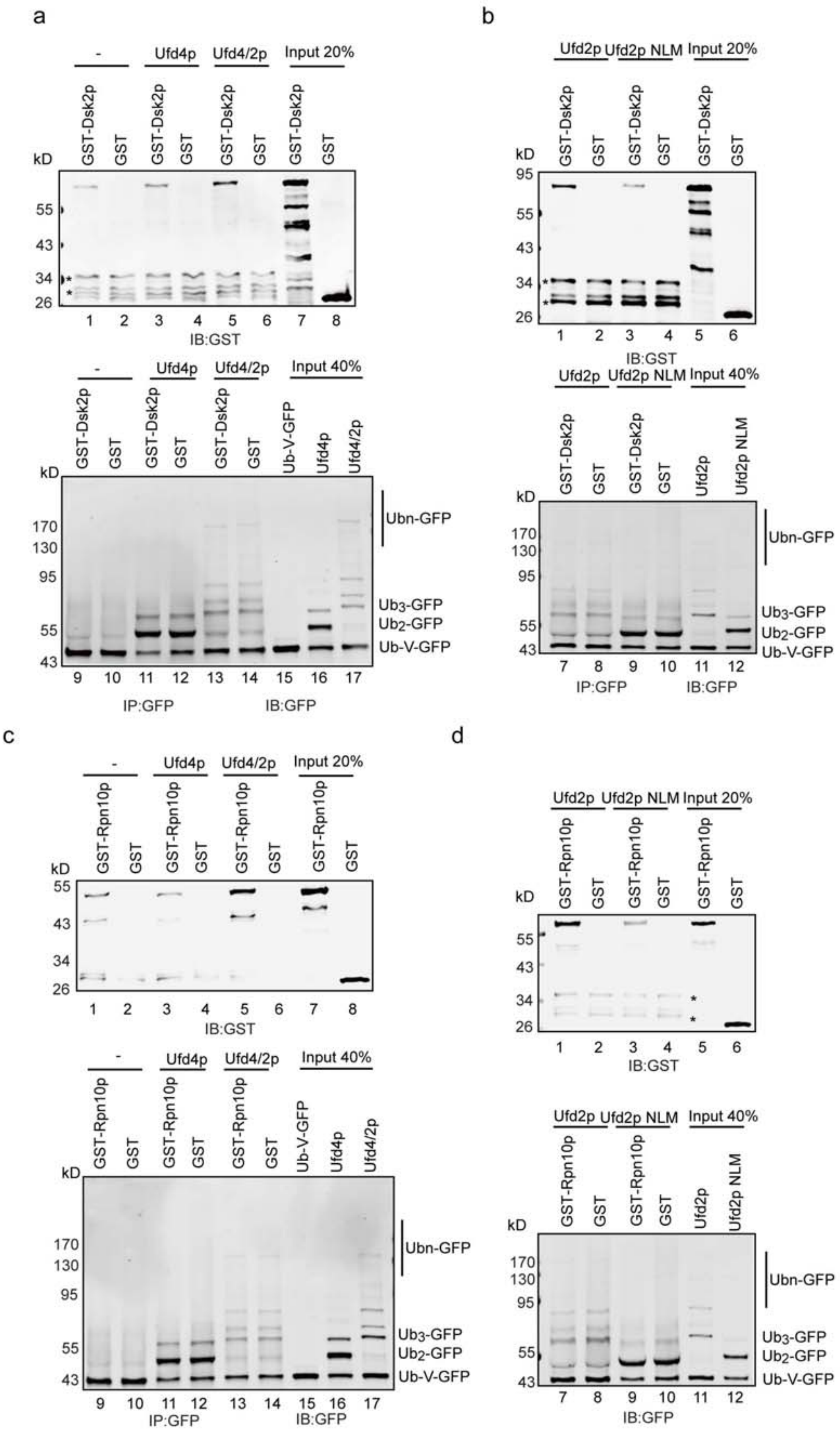

Supplementary Figure 12. Ub-V-GFP modified with branched ubiquitin chains can be recognized by Dsk2p and Rpn10p, Relate to Figure 5.

(a) and (c) Dsk2p and Rpn10p prefers to bind with branched ubiquitin chains modified Ub-V-GFP compare with those Lys29-linked ubiquitin chains modified Ub-V-GFP and Ub-V-GFP. Ub-V-GFP, Lys29-linked ubiquitin chain modified Ub-V-GFP (which was synthesized by Ufd4p) and branched ubiquitin chain modified Ub-V-GFP (which was synthesized by Udf4p-Ufd2p mediated ubiquitination) were immobilized on protein A-Sepharose beads by anti-GFP antibody, respectively. They were used to immunoprecipitate GST-Dsk2p, GST-Rpn10p and GST protein was used as a control group. The precipitated proteins were then analyzed by immunoblotting with the indicated antibodies. Asterisks indicate unspecific background signals from the IgG chains.

(b) and (d) Ufd2p NLM mediated ubiquitination products fails to be recognized by proteasome adaptor protein such as Dsk2p and Rpn10p. Wild-type Ufd2p and Ufd2p NLM mediated ubiquitination products were immobilized on protein A-Sepharose beads by anti-GFP antibody, respectively. They were used to immunoprecipitate GST-Dsk2p and GST-Rpn10p. Ubn-GFP which was modified by Ufd2p NLM mediated ubiquitination could not efficiently immunoprecipitate GST-Dsk2p and GST-Rpn10p. Asterisks indicate nonspecific background signals from the IgG chains.

Supplementary Figure 13

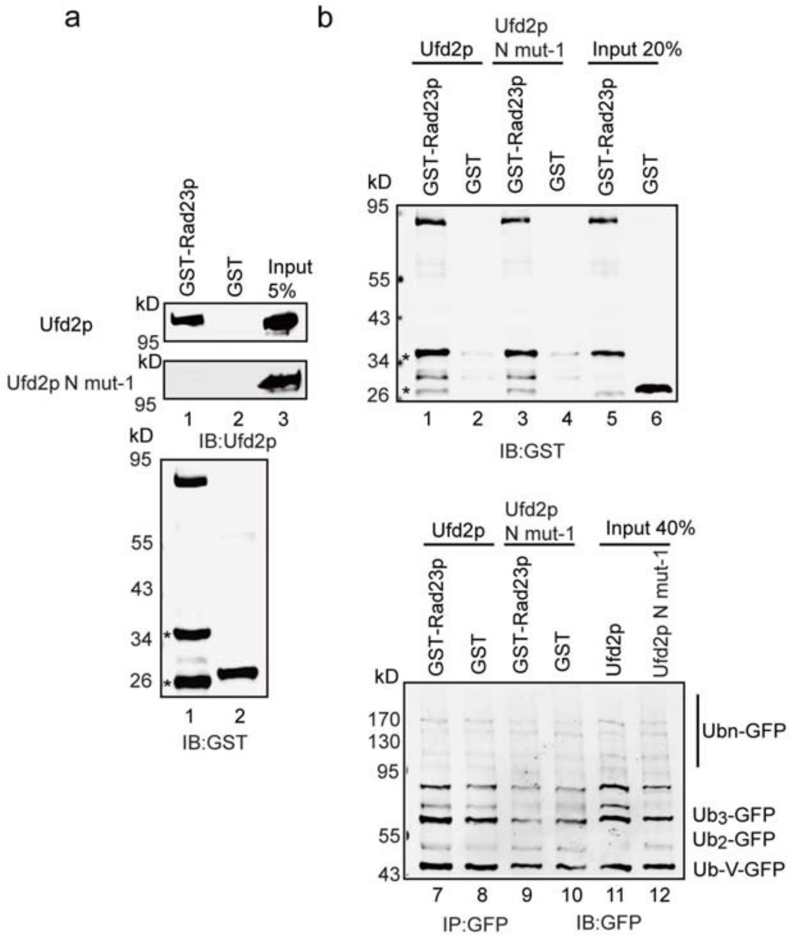

Supplementary Figure 13. Interaction between Rad23p and branched ubiquitin chain modified Ub-V-GFP is not intermediated by Ufd2p, Relate to Figure 5.

(a) Ufd2p N mut-1 could not bind to Rad23p. GST-Rad23p was used to pull down Ufd2p and Ufd2 N mut-1. GST protein was used as a control group.

(b) Branched ubiquitin chains modified Ub-V-GFP which was synthesized by the Rad23p binding defective Ufd2p N-mut1 protein was indistinguishable to those synthesized by wild-type Ufd2p. Branched ubiquitin chains modified Ub-V-GFP which was synthesized by either wild-type or the Rad23p binding defective Ufd2p N-mut1 was immobilized onto protein A-Sepharose beads by anti-GFP antibody. They were used to immunoprecipitate GST-Rad23p,

and GST protein was used as a control. The precipitated proteins were analyzed by immunoblotting with the indicated antibodies.

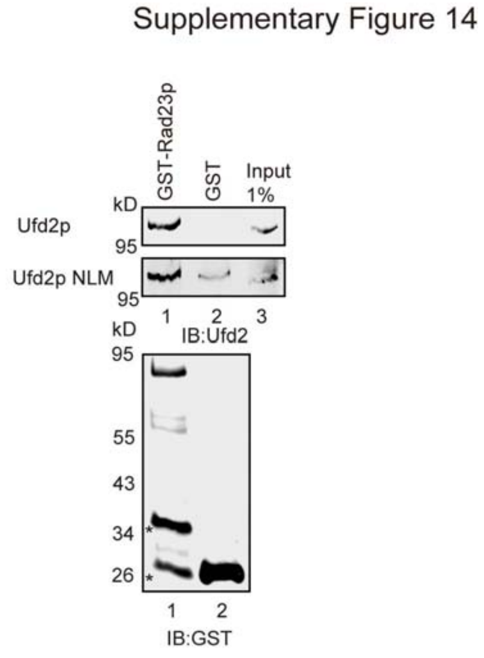

Supplementary Figure 14. Ufd2p NLM still could bind to Rad23p, Relate to Figure 5.

GST-Rad23p was used to pull down Ufd2p and Ufd2 NLM. GST protein was used as control.

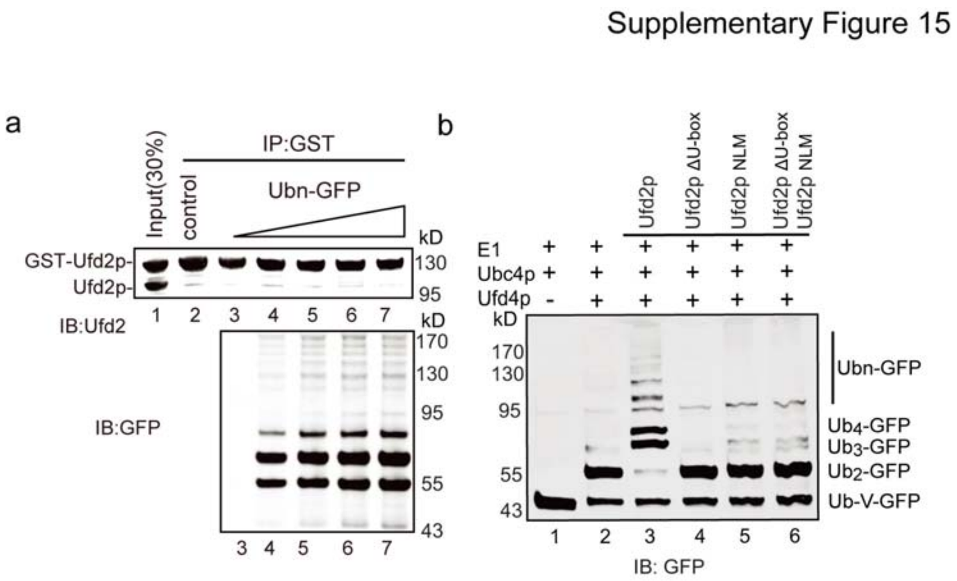

Supplementary Figure 15. Ufdp2 does not function as a dimer.

(a) Lys29-linked Ubn-GFP could not promote Ufd2p self-assembly. Equal amount of GST-tagged (GST-Ufd2p) and untagged Ufd2p were mixed in the presence of increasing amounts of Lys29-linked Ubn-GFP (lanes 3–7). GST-Ufd2p and its associated proteins were pulled down by GST beads and analyzed by immunoblotting with anti-Ufd2 antibody. Lane 1 shows input (30% of GST-Ufd2p and Ufd2p used in the binding experiment).

(b) Mixing Ufd2p ΔU-box and Ufd2p NLM mutants together could not restore any E3 liagase activity. Ufd2p, Ufd2p ΔU-box, Ufd2p NLM or mixture of the Ufd2p ΔU-box and Ufd2p NLM was incubated with E1, E2, Ufd4p, ATP, ubiquitin and Ub-V-GFP, and the products were detected using the anti-GFP antibody.

Supplementary Figure 16

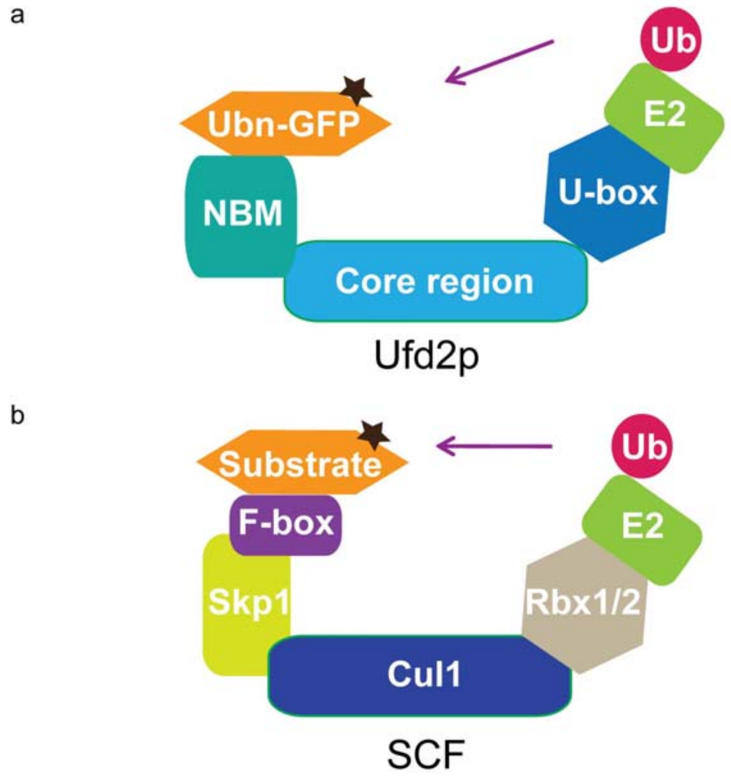

Supplementary Figure 16. Ufd2p catalyzes ubiquitination in a manner analogous to the SCF ubiquitin E3 ligase complex.

(a) A carton of Ufd2p mediated ubiquitination. Ufd2p contains three parts: the core region, the U-box domain and the N-terminal substrate binding motif (NBM). During the reaction, the U-box domain binds to E2~Ub and NBM recognizes the Lys29-linked Ub2-GFP, U-box domain then transfers Ub from E2 to Ub2-GFP.

(b) A carton of SCF mediated ubiquitination. The SCF E3 ligase contains the Cullin subunit which works as molecular scaffolds, its carboxyl terminus interacts with a RING-finger protein, its N-terminal region interacts with Skp1. which in turn, binds to F-box-proteins. During the reaction, the RING-finger protein interacts with E2~Ub, and the F-box-protein recruits substrate to the complex, and finally the ubiquitin could be directly transferred from E2 to the substrate.

Supplementary Tables

Supplementary Table 1. SPR parameters of GST-Ufd2p and GST-Ufd2p NLM binding with Ub-V-GFP

	ka(1/Ms)	kd(1/s)	KA(1/M)	KD(μM)
**Ufd2p::Ub-V-GFP**	784	1.99e-3	3.95e5	2.53
**Ufd2p NLM::Ub-V-GFP**	2.49e3	5.73e-3	4.34e5	2.31

Supplementary Table 2. Strains Used in This Study

Strain	Genotype	Source
By4742	*MATalpha, his3-Δ1, leu2-Δ0,lys2-Δ0, ura3-Δ0*	[1]
ufd2Δ	*MATalpha, his3-Δ1, leu2-Δ0,lys2-Δ0, ura3-Δ0,ufd2Δ::kanMX4*	[2]
SF10	*MATa ura3-52 trp1, lys2-801, leu2Δ1 pep4Δ::HIS3 prb1Δ1.6R can1*	[3]
LW0238	*MATalpha, his3-Δ1, leu2-Δ0::pESC-Ub-V-GFP-His6-FLAG::LEU2,lys2-Δ0, ura3-Δ0::pYC2::URA3*	This study
LW0239	*MATalpha, his3-Δ1, leu2-Δ0::pESC-Ub-V-GFP-His6-FLAG::LEU2,lys2-Δ0, ura3-Δ0::pYC2::URA3,ufd2Δ::kanMX4*	This study

LW0240	*MATalpha, his3-Δ1, leu2-Δ0::pESC-Ub-V-GFP-His6-FLAG::LEU2,lys2-Δ0, ura3-Δ0::pYC2-UFD2::URA3,ufd2Δ::kanMX4*	This study
LW0241	*MATalpha, his3-Δ1, leu2-Δ0::pESC-Ub-V-GFP-His6-FLAG::LEU2,lys2-Δ0, ura3-Δ0::pYC2-UFD2 NLM::URA3,ufd2Δ::kanMX4*	This study
LW0218	*MATalpha, his3-Δ1, leu2-Δ0::pESC::LEU2,lys2-Δ0, ura3-Δ0::pYes2-4×Myc-HMG2::URA3*	This study
LW0219	*MATalpha, his3-Δ1, leu2-Δ0::pESC::LEU2,lys2-Δ0, ura3-Δ0::pYes2-4×Myc-HMG2::URA3,ufd2Δ::kanMX4*	This study
LW0220	*MATalpha, his3-Δ1, leu2-Δ0::pESC-UFD2::LEU2,lys2-Δ0, ura3-Δ0::pYes2-4×Myc-HMG2::URA3,ufd2Δ::kanMX4*	This study
LW0221	*MATalpha, his3-Δ1, leu2-Δ0::pESC-UFD2 ΔU-box::LEU2,lys2-Δ0, ura3-Δ0::pYes2-4×Myc-HMG2::URA3,ufd2Δ::kanMX4*	This study
LW0222	*MATalpha, his3-Δ1, leu2-Δ0::pESC-UFD2 NLM::LEU2,lys2-Δ0, ura3-Δ0::pYes2-4×Myc-HMG2::URA3,ufd2Δ::kanMX4*	This study
LW0223	*MATalpha, his3-Δ1, leu2-Δ0::pESC::LEU2,lys2-Δ0, ura3-*	This study

	Δ0::pYes2-9×Myc-SPT23::URA3	
LW0224	*MATalpha, his3-Δ1, leu2-Δ0::pESC::LEU2,lys2-Δ0, ura3-Δ0::pYes2-9×Myc-SPT23::URA3,ufd2Δ::kanMX4*	This study
LW0225	*MATalpha, his3-Δ1, leu2-Δ0::pESC-UFD2::LEU2,lys2-Δ0, ura3-Δ0::pYes2-9×Myc-SPT23::URA3,ufd2Δ::kanMX4*	This study
LW0226	*MATalpha, his3-Δ1, leu2-Δ0::pESC-UFD2 ΔU-box::LEU2,lys2-Δ0, ura3-Δ0::pYes2-9×Myc-SPT23::URA3,ufd2Δ::kanMX4*	This study
LW0227	*MATalpha, his3-Δ1, leu2-Δ0::pESC-UFD2 NLM::LEU2,lys2-Δ0, ura3-Δ0::pYes2-9×Myc-SPT23::URA3,ufd2Δ::kanMX4*	This study
LW0315	*MATalpha, his3-Δ1::pRS313::HIS3, leu2-Δ0,lys2-Δ0, ura3-Δ0*	This study
LW0316	*MATalpha, his3-Δ1::pRS313::HIS3, leu2-Δ0,lys2-Δ0, ura3-Δ0,ufd2Δ::kanMX4*	This study
LW0317	*MATalpha, his3-Δ1::pRS313-pUFD2-UFD2::HIS3, leu2-Δ0,lys2-Δ0, ura3-Δ0,ufd2Δ::kanMX4*	This study
LW0318	*MATalpha, his3-Δ1::pRS313-pUFD2-UFD2 ΔU-box::HIS3, leu2-Δ0,lys2-Δ0, ura3-Δ0,ufd2Δ::kanMX4*	This study

LW0319	*MATalpha, his3-Δ1::pRS313-pUFD2-UFD2 NLM::HIS3, leu2-Δ0,lys2-Δ0, ura3-Δ0,ufd2Δ::kanMX4*	This study
LW0320	*MATalpha, his3-Δ1, leu2-Δ0::pESC-Myc-Ub53TEV-FLAG-Ub64TEV/FLAG::LEU2,lys2-Δ0, ura3-Δ0::pYEP-Ub-V-GFP-His6::URA3*	This study
LW0321	*MATalpha, his3-Δ1, leu2-Δ0::pESC-Myc-Ub53TEV-FLAG-Ub64TEV/FLAG::LEU2,lys2-Δ0, ura3-Δ0::pYEP-Ub-V-GFP-His6::URA3,ufd2Δ::kanMX4*	This study
LW0322	*MATalpha, his3-Δ1, leu2-Δ0::pESC-Myc-Ub53TEV-FLAG-Ub64TEV/FLAG::LEU2,lys2-Δ0, ura3-Δ0::pYEP-K29RUb-V-GFP-His6::URA3*	This study
LW0323	*MATalpha, his3-Δ1, leu2-Δ0::pESC-Myc-Ub53TEV-FLAG-Ub64TEV/FLAG::LEU2,lys2-Δ0, ura3-Δ0::pYEP-K48RUb-V-GFP-His6::URA3*	This study
LW0324	*MATalpha, his3-Δ1, leu2-Δ0::pESC-Myc-Ub53TEV-FLAG-Ub64TEV/FLAG::LEU2,lys2-Δ0, ura3-Δ0::pYEP-K29/48RUb-V-GFP-His6::URA3*	This study

Supplementary Table 3. Plasmids Used in This Study

Plasmid	Description	Source
pLW0160	His6-Ufd2 in pET28a	[4]
pLW0173	His6-Ubc4 in pET28a	[4]
pLW0527	GFP-His6 in pET28a	This study
pLW0273	Ub-V-GFP-His6 in pET21a	[5]
pLW0341	K6R Ub-V-GFP-His6 in pET21a	This study
pLW0342	K11R Ub-V-GFP-His6 in pET21a	This study
pLW0343	K27R Ub-V-GFP-His6 in pET21a	This study
pLW0344	K29R Ub-V-GFP-His6 in pET21a	This study
pLW0345	K33R Ub-V-GFP-His6 in pET21a	This study
pLW0346	K48R Ub-V-GFP-His6 in pET21a	This study
pLW0347	K63R Ub-V-GFP-His6 in pET21a	This study
pLW0348	K0 Ub-V-GFP-His6 in pET21a	This study
pLW0349	K6 only Ub-V-GFP-His6 in pET21a	This study
pLW0350	K11 only Ub-V-GFP-His6 in pET21a	This study

pLW0351	K27only Ub-V-GFP-His6 in pET21a	This study
pLW0352	K29 only Ub-V-GFP-His6 in pET21a	This study
pLW0353	K33 only Ub-V-GFP-His6 in pET21a	This study
pLW0354	K48 only Ub-V-GFP-His6 in pET21a	This study
pLW0355	K63 only Ub-V-GFP-His6 in pET21a	This study
pLW0356	K29/48 only Ub-V-GFP-His6 in pET21a	This study
pEV0027	His6-FLAG-Ubiquitin in pET28a	This study
pEV0031	His6-FLAG-K29R Ubiquitin in pET28a	This study
pEV0033	His6-FLAG-K48R Ubiquitin in pET28a	This study
pLW0252	His6-FLAG-K29/48R Ubiquitin in pET28a	This study
pEV0034	His6-FLAG-K0 Ubiquitin in pET28a	This study
pEV0035	His6-FLAG-K6 only Ubiquitin in pET28a	This study
pEV0036	His6-FLAG-K11 only Ubiquitin in pET28a	This study
pEV0037	His6-FLAG-K27 only Ubiquitin in pET28a	This study
pEV0038	His6-FLAG-K29 only Ubiquitin in pET28a	This study

pEV0039	His6-FLAG-K33 only Ubiquitin in pET28a	This study
pEV0040	His6-FLAG-K48only Ubiquitin in pET28a	This study
pEV0041	His6-FLAG-K63 only Ubiquitin in pET28a	This study
pLW0100	His6-Ub-D77 in pET3a	[6]
pLW0253	His6-K48R Ub-D77 in pET3a	This study
pLW0102	His6-Yuh1 in pET28a	[6]
pEV0042	His6-Ufd2 ΔU-box in pET28a	[4]
pEV0468	GST-Ufd2 in pGEX-4t-1	This study
pEV0451	GST-Ufd2 CF1 (519-961aa) in pGEX-4t-1	This study
pEV0441	GST-Ufd2 CF2 (760-961aa) in pGEX-4t-1	This study
pEV0442	GST-Ufd2 CF3 (879-961aa) in pGEX-4t-1	This study
pEV0483	GST-Ufd2 MFD1(Δ111-879aa) in pGEX-4t-1	This study
pEV0476	GST-Ufd2 MFD2(Δ75-879aa) in pGEX-4t-1	This study
pEV0471	GST-Ufd2 MFD3(Δ55-879aa) in pGEX-4t-1	This study
pEV0481	GST-Ufd2 MFD4(Δ55-75&111-879aa) in pGEX-4t-1	This study

pEV0464	GST-Ufd2 Ufd2 NHD1(Δ35-55aa) in pGEX-4t-1	This study
pEV0462	GST-Ufd2 MFD2(Δ75-111aa) in pGEX-4t-1	This study
pEV0447	GST-Ufd2 35-961aa in pGEX-4t-1	This study
pEV0449	GST-Ufd2 111-961aa in pGEX-4t-1	This study
pEV0489	GST-Ufd2 N mut-1(L44A Y97A V100A I104A F107A) in pGEX-4t-1	This study
pEV0490	GST-Ufd2 N mut-2(L44A Y97A V100A F107A L93A G96A) in pGEX-4t-1	This study
pEV0491	GST-Ufd2 N mut-3(L44A Y97A V100A I104A F107A L93A G96A) in pGEX-4t-1	This study
pEV0555	GST-Ufd2 Δ103-111aa in pGEX-4t-1	This study
pEV0591	GST-Ufd2 51-55mut(E51A K52A L53A D54A K55A) in pGEX-4t-1	This study
pEV0592	GST-Ufd2 51-55&108-111 mut (E51A K52A L53A D54A K55A M109A N110A) in pGEX-4t-1	This study
pEV0588	GST-Ufd2 51-55&Δ103-111 mut (E51A K52A L53A D54A K55A&Δ103-111aa) in pGEX-4t-1	This study

pEV0720	GST-Ufd2 1-111aa in pGEX-4t-1	This study
pEV0721	GST-Ufd2 1-111aa NLM (E51A K52A L53A D54A K55A E105A N106A M109A N110A) in pGEX-4t-1	This study
pEV0603	GST-Ufd2 NLM (E51A K52A L53A D54A K55A E105A N106A M109A N110A) in pGEX-4t-1	This study
pEV0308	GST-Rad23 in pGEX-4t-1	This study
pEV0307	GST-Dsk2 in pGEX-4t-1	This study
pEV0306	GST-Rpn10 in pGEX-4t-1	This study
pEV0688	Ub-V-GFP-His6-FLAG in pESC-LEU, LEU2	This study
pLW0161	Xpress-His6-Ufd2 in pYC2-NT,URA3	This study
pEV0627	Xpress-His6-Ufd2 NLM in pYC2-NT,URA3	This study
pEV0512	4×myc-Hmg2 in pYES2,URA3	This study
pEV0511	9×myc-Spt23 in pYES2,URA3	This study
pEV0730	Ufd2 in pESC-LEU,LEU2	This study
pEV0731	Ufd2 ΔU-box in pESC-LEU,LEU2	This study

pEV0732	Ufd2 NLM in pESC-LEU,LEU2	This study
pEV0752	pUFD2-Ufd2 in pRS313,HIS3	This study
pEV0753	pUFD2-Ufd2 ΔU-box in pRS313,HIS3	This study
pEV0754	pUFD2-Ufd2 NLM in pRS313,HIS3	This study
pLW0294	Xpress-His6-Ufd4 in pYC2-NT,URA3	This study
pEV0756	His6-FLAG-Ubiquitin G53TEV in pET28a	This study
pEV0757	His6-FLAG-Ubiquitin E64TEV/FLAG in pET28a	This study
pEV0758	Myc-Ubiquitin G53TEV/FLAG-Ubiquitin E64TEV/FLAG in pESC-LEU,LEU2	This study
pEV0759	Ub-V-GFP-His6 in pYEP,URA3	This study
pEV0760	K29R Ub-V-GFP-His6 in pYEP,URA3	This study
pEV0761	K48R Ub-V-GFP-His6 in pYEP,URA3	This study
pEV0762	K29/48R Ub-V-GFP-His6 in pYEP,URA3	This study
pEV0763	GST-Ufd3 in pGEX-4t-1	This study
pEV0764	GST-Otu1 in pGEX-4t-1	This study

pEV0765	Ubc1 in pET28a	This study
pEV0766	Ubc2 in pET28a	This study
pEV0767	Ubc3 in pET28a	This study
pEV0768	Ubc4 in pET28a	This study
pEV0769	Ubc5 in pET28a	This study
pEV0770	Ubc6 ΔTM in pET28a	This study
pEV0771	Ubc7 in pET28a	This study
pEV0772	Ubc8 in pET28a	This study
pEV0773	Ubc10 in pET28a	This study
pEV0774	Ubc11 in pET28a	This study
pEV0775	GST-Ubc13 in pGEX-4t-1	This study
pEV0776	Mms2 in pET28a	This study
pEV0777	GST-Tom1 CT in pGEX-4t-1	This study
pEV0778	GST-Rsp5 in pGEX-4t-1	This study
pEV0779	GST-Hul4 in pGEX-4t-1	This study

pEV0780	GST-Hul5 in pGEX-4t-1	This study
pLW0001	His6-Ube2g2 in pET28a	[7]
pLW0030	GST- gp78 C in pGEX-4t-1	[7]
pLW0308	Ub4-V-GFP-His6 in pET21a	This study
pLW0309	Ub2-V-GFP-His6 in pET21a	This study
pLW0341	K6R-Ub-V-GFP-His6 in pET21a	This study
pLW0342	K11R-Ub-V -GFP-His6 in pET21a	This study
pLW0343	K27R-Ub-V -GFP-His6 in pET21a	This study
pLW0344	K29R-Ub-V -GFP-His6 in pET21a	This study
pLW0345	K33R-Ub-V -GFP-His6 in pET21a	This study
pLW0346	K48R-Ub-V -GFP-His6 in pET21a	This study
pLW0347	K63R-Ub-V -GFP-His6 in pET21a	This study

Supplemental references

1. Brachmann CB*, et al.* Designer deletion strains derived from Saccharomyces cerevisiae S288C: a useful set of strains and plasmids for PCR-mediated gene disruption and other applications. *Yeast* **14**, 115-132 (1998).

2. Ye Y, Meyer HH, Rapoport TA. The AAA ATPase Cdc48/p97 and its partners transport proteins from the ER into the cytosol. *Nature* **414**, 652-656 (2001).

3. Holmes SG*, et al.* Hyperactivation of the silencing proteins, Sir2p and Sir3p, causes chromosome loss. *Genetics* **145**, 605-614 (1997).

4. Tu D, Li W, Ye Y, Brunger AT. Structure and function of the yeast U-box-containing ubiquitin ligase Ufd2p. *Proc Natl Acad Sci U S A* **104**, 15599-15606 (2007).

5. Saeki Y, Tayama Y, Toh-e A, Yokosawa H. Definitive evidence for Ufd2-catalyzed elongation of the ubiquitin chain through Lys48 linkage. *Biochemical and biophysical research communications* **320**, 840-845 (2004).

6. Raasi S, Pickart CM. Ubiquitin chain synthesis. *Methods Mol Biol* **301**, 47-55 (2005).

7. Li W*, et al.* Mechanistic insights into active site-associated polyubiquitination by the ubiquitin-conjugating enzyme Ube2g2. *Proc Natl Acad Sci U S A* **106**, 3722-3727 (2009).
